# Structural and functional insights into the lipopolysaccharide ABC transporter LptB_2_FG

**DOI:** 10.1038/s41467-017-00273-5

**Published:** 2017-08-09

**Authors:** Haohao Dong, Zhengyu Zhang, Xiaodi Tang, Neil G. Paterson, Changjiang Dong

**Affiliations:** 1 State Key Laboratory of Biotherapy and Cancer Center, West China Hospital, Sichuan University and Collaborative Innovation Center of Biotherapy, Chengdu, 610041 China; 2 Biomedical Research Centre, Norwich Medical School, University of East Anglia, Norwich Research Park, Norwich, NR4 7TJ UK; 3 Kennedy Institute of Rheumatology, Nuffield Department of Orthopaedics, Rheumatology and Musculoskeletal Sciences, University of Oxford, Oxford, OX3 7FY UK; 4 Diamond Light Source, Harwell Science and Innovation Campus, Didcot, Oxfordshire OX11 0DE UK

## Abstract

The cell surface of most Gram-negative bacteria contains lipopolysaccharide that is essential for their viability and drug resistance. A 134-kDa protein complex LptB_2_FG is unique among ATP-binding cassette transporters because it extracts lipopolysaccharide from the external leaflet of the inner membrane and propels it along a filament that extends across the periplasm to directly deliver lipopolysaccharide into the external leaflet of the outer membrane. Here we report the crystal structure of the lipopolysaccharide transporter LptB_2_FG from *Klebsiella pneumoniae*, in which both LptF and LptG are composed of a β-jellyroll-like periplasmic domain and six α-helical segments in the transmembrane domain. LptF and LptG form a central cavity containing highly conserved hydrophobic residues. Structural and functional studies suggest that LptB_2_FG uses an alternating lateral access mechanism to extract lipopolysaccharide and traffic it along the hydrophobic cavity toward the transporter’s periplasmic domains.

## Abstract (web summary)

Lipopolysaccharides (LPS) are synthesized at the periplasmic side of the inner membrane of Gram-negative bacteria and are then extracted by the LptB_2_FG complex during the first step of LPS transport to the outer membrane. Here the authors present the LptB_2_FG structure, which supports an alternating lateral access mechanism for LPS extraction.

## Introduction

**Fig. 1:**
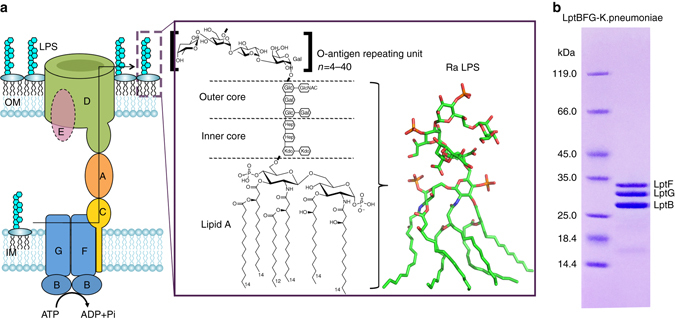
LPS transport from the IM to the OM by the trans-envelope complex LptABCDEFG. **a** LPS is extracted from the periplasmic side of the IM by the ABC transporter LptB_2_FG, and is delivered to an IM protein LptC, which forms a complex with LptB_2_FG. LptC composes of a single membrane spanning domain and a large periplasmic domain, which forms a periplasmic bridge with LptA and the N-terminal domain of LptD. LPS is then inserted into the OM by LptD/E complex. LPS contains O-antigen, core oligosaccharide, and lipid A components, of which the O-antigen has 4-40 O-antigen repeat units. Ra-LPS, rough LPS, Kdo, 3-deoxy-D-manno-oct-2-ulosonic acid, Hep, L-glycero-D-manno-heptose, Glc, D-glucose, Gal, D-galactose. **b** Coomassie brilliant blue staining of the purified *K. pneumoniae* LptB_2_FG

The asymmetric outer membrane (OM) comprises phospholipids in the inner leaflet and lipopolysaccharide (LPS) in the outer leaflet and is essential for Gram-negative bacteria^1–3^. In particular, LPS plays a critical role in drug resistance and pathogenesis^4, 5^. LPS typically contains three components: lipid A, core oligosaccharide, and O-antigen polysaccharide (Fig. 1a). The O-antigen can contain up to 200 sugars, which contribute to bacterial resistance to the host immune system^4, 5^. The lipid A and the core oligosaccharide are synthesized at the cytoplasmic side of the inner membrane (IM) and then flipped to the periplasmic side by MsbA^6^. The precursor of the O-antigen units are synthesized independently in the cytoplasm and transported by Wzx into the periplasmic side of the IM, where they are polymerized and ligated to lipid A core oligosaccharide by WaaL to form LPS^1, 2^. Transporting millions of these large amphipathic LPS molecules from the IM across the aqueous periplasm and correctly inserting them into the outer leaflet of the hydrophobic OM during cell division is a formidable challenge^1, 7^.

Seven LPS transport proteins, LptA-G, are responsible for transporting LPS from the IM to the cell surface^8–12^ (Fig. 1a). Structural and functional studies reveal that the carboxyl (C) terminus of LptC interacts with the amino (N) terminus of LptA, and that the C terminus of LptA interacts with the N terminus of LptD forming a bridge to transport LPS across the periplasm^9, 12–15^. The two-protein complex, LptD/E^11, 16–21^, catalyzes the insertion of LPS into the OM through the intramembranous barrel and lateral opening of LptD^22–25^. The ABC transporter LptB_2_FG complex has a molecular weight of ∼134 kDa, and contains two transmembrane domains (TMDs), LptF and LptG, and two nucleotide-binding domains (NBDs), LptB_2_^8, 26^ (Fig. 1a, b). All three components of LptB_2_FG are essential for LPS transport in *Escherichia coli* ^8, 10, 12^. LptB hydrolyzes ATP to provide energy for LPS extraction and transport^27^. Unlike other ABC transporters which transport their substrates across the membrane, the LPS transporter LptB_2_FG extracts LPS from the periplasmic side of the IM and passes it to the periplasmic domain of the IM protein LptC^7^. However, the LPS extraction mechanism remains unknown.

To understand the structural basis of how ABC transporter LptB_2_FG extracts and transports LPS, we have solved the crystal structure of the LptB_2_FG complex from *Klebsiella pneumoniae*. Functional assays on *lptFG*-depleted *E. coli* NR1113 strain^8^ suggest that the transporter LptB_2_FG extracts LPS from the periplasmic leaflet of the IM to the transporter’s internal cavity and reorients it toward the periplasmic domain of LptF or LptG. These features are different from other structurally characterized ABC transporters.

## Results

### Purified LptB_2_FG has ATPase activity

The purified LptB_2_FG transporters have ATPase activity (Supplementary Fig. 1), and our functional assay showed that the His-, Flag-, and Myc-tagged LptB_2_FG can complement *lptFG*-depleted strain NR1113 (Supplementary Fig. 2). Both the native *lptF* and *lptG* genes have been deleted from *E. coli* NR1113, but they are covered by an arabinose-inducible copy of the lptFG operon at the λatt site. When LptF and LptG are depleted by omitting the inducer arabinose from the growth medium bacterial growth ceases^8^. The kanamycin-resistant plasmid pTRC99a-Kan containing lptBFG was used as the template for lptBFG mutagenesis and expression to complement bacterial growth of *E. coli* NR1113 (see “Methods” section). These data are consistent with the finding that LptB with the C-terminal His tag displays its ATPase activity in vitro^28^ and can rescue *lptB*-depleted *E. coli* cells^27^.

### Overall structure of LptB_2_FG

LptB_2_FG of *K. pneumoniae* was expressed, purified (Fig. 1b), and crystallized (see “Methods” section). The crystals belong to space group *I*2_1_2_1_2_1_ with the cell dimensions *a* = 105.25 Å, *b* = 210.52 Å, *c* = 258.94 Å, and *α* = *β* = *γ* = 90°. Heavy-atom derivatives were obtained by soaking the crystals in potassium tetranitroplatinate (II) (see “Methods” section). The structure was determined to a resolution of 3.7 Ångström (Å) by the single-wavelength anomalous dispersion (SAD) using data sets collected at 1.0723 Å, and the sequence register was validated using sulfur anomalous data and selenomethionine anomalous data (Supplementary Fig. 3 and Table 1). There is one *K. pneumoniae* LptB_2_FG transporter molecule per asymmetric unit (Supplementary Figs. 3 and 4); the solvent content of the crystals was 77%, which helped to generate a clear experimental electron density map (Supplementary Fig. 5). Details of the structure determination and the model building are provided in the “Methods” section.

**Table 1:** Data collection and refinement statistics

	LptBFG-Pt	LptBFG-Hg	LptBFG-sulfur	LptBFG-Se^a^
*Data collection* ^b^				
Space group	*I*2_1_2_1_2_1_	*I*2_1_2_1_2_1_	*I*2_1_2_1_2_1_	*P*2_1_2_1_2_1_
Cell dimensions				
*a*, *b*, *c* (Å)	105.3, 210.5, 258.9	100.9, 215.9, 258.6	106.9, 212.1, 260.6	110.15, 124.53, 398.09
α, β, γ (°)	90.0, 90.0, 90.0	90.0, 90.0, 90.0	90.0, 90.0, 90.0	90.0, 90.0, 90.0
Wavelength (Å)	1.07226	1.00068	1.77120	0.9795
Resolution (Å)	29.96–3.70 (3.90–3.70)	22.99–5.14 (5.27–5.14)	29.15–4.23 (4.64–4.23)	29.96–6.00 (6.71–6.00)
*R*_merge_ (%)	26.2 (>100.0)	40.9 (>100.0)	21.1 (72.4)	22.0 (>100.0)
*CC*_1/2_ (%)	100 (94.6)	99.5 (76.0)	100 (99.6)	99.8 (84.9)
*I*/σ(*I*)	15.5 (1.7)	6.0 (1.1)	26.4 (7.9)	10.7 (1.5)
Completeness (%)	99.8 (99.8)	99.0 (99.3)	97.3 (90.1)	99.2 (100.0)
Redundancy	120.8 (117.5)	26.2 (27.9)	173.6 (81.8)	19.4 (20.2)
*Phasing*				
Resolution (Å)	29.96–3.70			
Sites (Pt)	4			
Figure of merit	0.332			
*Refinement*				
Resolution (Å)	29.96–3.70			
No. reflections	20311			
*R*_work_/*R*_free_	0.29/0.32			
No. atoms				
Protein	8422			
Ligand/ion	2			
Water	0			
*B*-factors				
Protein	112.40			
R.m.s. deviations				
Bond lengths (Å)	0.009			
Bond angles (°)	1.160			
Ramachandran statistics				
Allowed (%)	96.8			
Outliers (%)	3.2			
PDB code	5L75			

^a^The SeMet crystals of LptB_2_FG were from *Shigella flexneri*

^b^Statistics for data collection are those prior to anisotropic correction. Data were truncated along the surface defined by *I*/σ(*I*) = 1.2 using the STARANISO web server. These corrected data were used for subsequent phasing and refinement

**Fig. 2:**
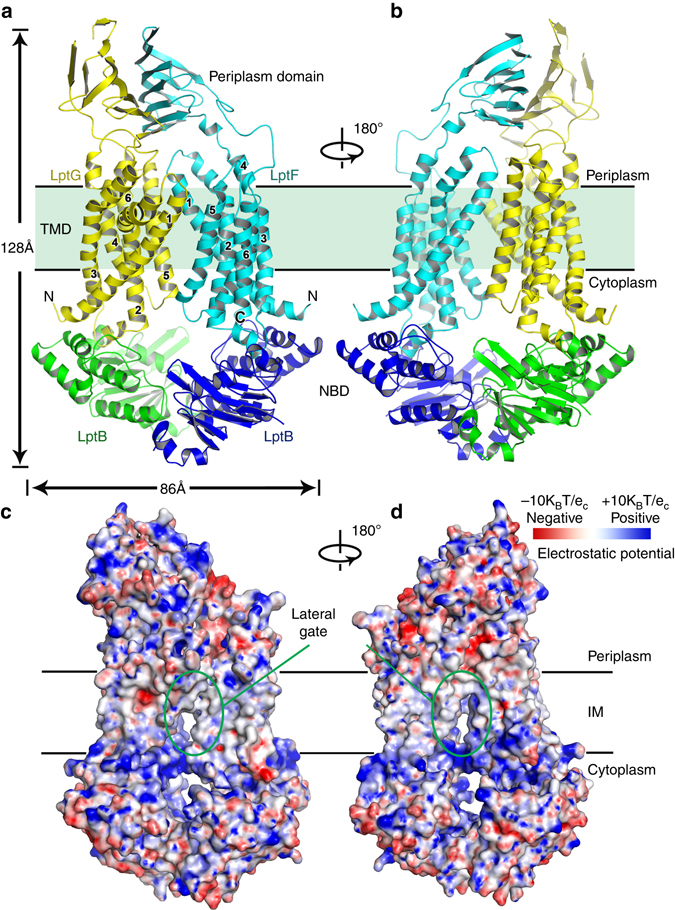
Crystal structure of the *K. pneumoniae* lipopolysaccharide transporter LptB_2_FG. **a** Cartoon representation of LptB_2_FG. LptF, LptG, and the two LptB molecules are shown in *cyan*, *yellow*, *green*, and *blue*, respectively. **b** The LptB_2_FG complex rotated by 180° along the *y*-axis relative to the *left panel*. **c** The electrostatic potential map of LptB_2_FG. There is a lateral gate between TM1G and TM5F. The most positive potential is colored in *blue* and the most negative potential is colored *red*. **d** The LptB_2_FG electrostatic potential map rotated by 180° along the *y*-axis relative to the *left panel*. There is a lateral opening between TM1F and TM5G

LptF and LptG each contain six α-helical transmembrane segments, namely TM1-6-F and TM1-6-G, respectively, a periplasmic β-jellyroll domain, three periplasmic loops, and a pair of cytoplasmic turns (Fig. 2a, b). LptF and LptG form a hetero-dimeric cavity with a total of 12 TM segments and the two LptB copies form a homo-dimer in a “V” shape at the cytoplasmic face of the TMDs region (Fig. 2c, d). Overall, the dimension of the LptB_2_FG transporter is of ~86 Å in width and 128 Å in length. The two periplasmic β-jellyroll domains are shifted to one side of the transporter thus generating a periplasmic opening at the side of the TM5F-TM1G interface (Fig. 2a, b).

The Dali server^29^ search revealed that LptF and LptG are distinct compared to previously published structures. The closest entry with partial similarity to LptF or LptG is the sorting nexin 9 (PDB code 3DYT)^30^ with a Z score of 5.4, and a root mean squared deviation (RMSD) of 7.1 over 140-aligned Cα atoms. In addition, this ABC exporter has two large periplasmic domains not found in other ABC exporter structures.

The well-known ABC transporters, MalFGK_2_^31–33^, BtuCD-F^34–37^, PglK^38^, and MsbA^6^, transport maltose, vitamin B-12, lipid-linked oligosaccharide, and lipid A core oligosaccharide across the IM, respectively. In contrast, the ABC exporter LptB_2_FG does not transport its substrate across the IM and sheds light on this transport mechanism.

### Transporter LptB_2_FG has lateral gaps in the IM

**Fig. 3:**
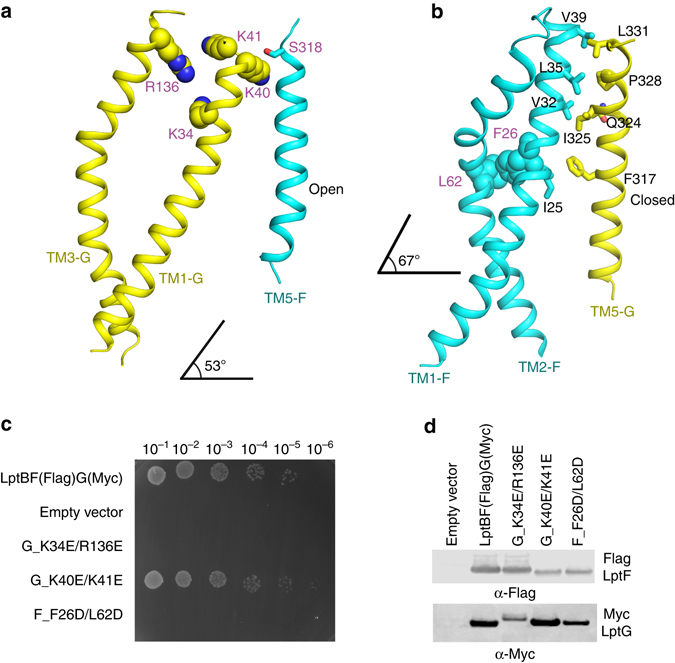
The transmembrane domains of LptB_2_FG and the lateral openings. The color scheme is the same as in Fig. 2. **a** The lateral gate of TM1G-5F interaction. The residues K40 of TM1G and S318 of TM5F forming the contact are shown in *sphere* and *stick*, respectively. The positively charged and highly conserved residues K34 and R136 of LptG are also shown in *sphere*. **b** The lateral gate of TM1F-5G interaction. There are five interactions within TM1F-5G (F_V32/G_I325, F_V32/G_Q324, F_V39/G_L331, F_L35/G_P328, and F_I25/G_ F317), shown in *stick*. The hydrophobic residues F26 and L62 of LptF are shown in *sphere*. The TM1F(G) crosses the IM at an angle of 67° and 53° to the membrane plane, respectively. **c** Functional assays of the double mutants LptG K34E/R136E, LptG K40E/K41E, and the LptF F26D/L62D in NR1113 depletion strain. NR1113 cells were transformed with the empty vector (pTRC99a_Kan, negative control) or the vector encoding LptBF(Flag)G(Myc) (positive control) or its mutant derivatives. All bacterial cultures were adjusted to an OD_600_ = 0.5 with fresh medium, serially diluted 1:10 as indicated on the *top* of the figure and then replica plated in agar plates containing kanamycin but in the absence of L-arabinose. Row 1: NR1113 cells transformed with LptBF(Flag)G(Myc) plasmid was used as a positive control. Row 2: NR1113 cells transformed with the empty plasmid (pTRC99a_Kan) was used as the negative control. Row 3: LptG double-mutant K34E/R136E. Row 4: LptG double-mutant K40E/K41E. Row 5: LptF double-mutant F26D/L62D. **d** Detection of protein expression levels of LptF(Flag)G(Myc) of the negative control, positive control, and the mutants of G_K34E/R136E, G_K40E/K41E, and F_F26D/L62D by western blotting. The bacterial cells for western blotting were cultured in the presence of 0.2% L-arabinose

LptF and LptG each form half of the transporter in the IM by contributing TM1-6, with TM1 of each subunit interfacing with TM5 of the other, approximating with twofold rotational symmetry (Fig. 2a, b). TM1F(G) crosses the IM at an angle of ~67° or 53° to the membrane plane (Fig. 3a, b). This feature separates TM1F-5G and TM5F-1G at their base, leaving visible gaps in the electrostatic potential surface map between interfacing segments (Fig. 2c, d), forming the lateral gates of the transporter LptB_2_FG. There are five interactions within TM1F-5G (F_V32/G_I325, F_V32/G_Q324, F_V39/G_L331, F_L35/G_P328, and F_I25/G_ F317) and one interaction within TM5F-1G on the periplasmic side (F_S318/G_K40) (Fig. 3a, b), indicating that the lateral gate TM1F-5G is in a closed form and the lateral gate TM5F-1G is in an open form.

### The cavity of LptB_2_FG

**Fig. 4:**
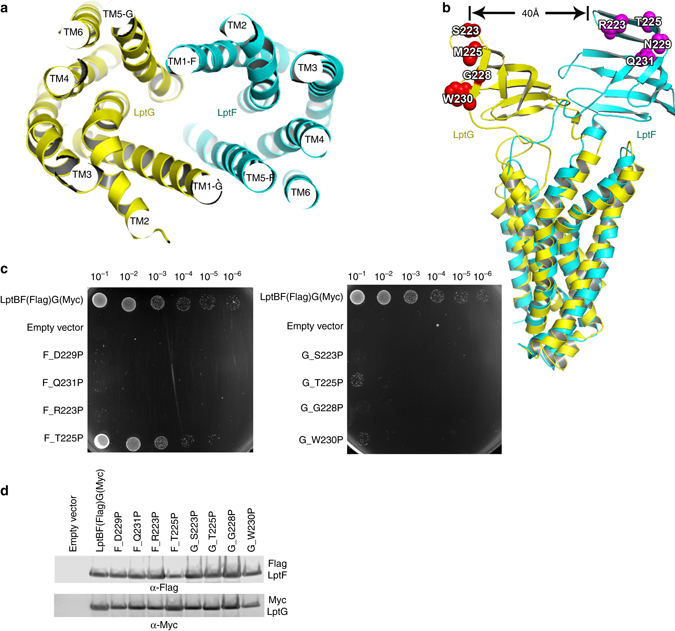
LptB2FG cavity and the periplasmic domains. The color scheme is the same as in Fig. 2. **a** The transmembrane domains of LptB_2_FG, *top view*. Both LptF and LptG have six TM segments respectively, forming a cavity. **b** The superimposition of LptF and LptG from *K. pneumoniae*. The proline substitutions are shown in sphere. **c** Functional assays of the single proline mutants of periplasmic domains of LptF and LptG according to *E. coli* amino-acid sequences. NR1113 cells were transformed with empty vector (pTRC99a_Kan, negative control) or the vector encoding LptBF(Flag)G(Myc) (positive control) or its single proline mutant derivatives. All bacterial cultures were adjusted to an OD_600_ nm = 0.5 with fresh medium, serially dilution 1:10 as indicated on the *top* of the figure and then replica plated in agar plates containing kanamycin but in the absence of L-arabinose. The positive control (lptBF(Flag)G(Myc) in NR1113), the negative control (empty plasmid in NR1113), LptF mutants F_D229P, F_Q231P, F_R223P and F_T225P, and LptG mutants G_S223P, G_T225P, G_G228P, and G_W230P were used in the functional assays. Double proline substitutions (F_D229P/Q231P, F_T225P/R223P, G_G228P/W230P, and G_T225P/S223P) are reported in Supplementary Fig. 2. **d** The protein expression levels of the mutants shown by the western blotting. The LptF and LptG protein expression of the negative control (empty plasmid), the positive control (plasmid with LptBF(Flag)G(Myc)), LptF mutants D229P, F_Q231P, F_R223P, and F_T225P, and LptG mutants G_S223P, G_T225P, G_G228P, and G_W230P were detected. The bacteria cells for western blotting were cultured in the presence of 0.2% L-arabinose. All mutants in Figs. 3 and 4 and Supplementary Fig. 2 are based on *E. coli* amino-acid sequence. The difference in residues is listed here: LptF-D229 (N229_*K. pneumoniae*), LptG-T225 (M225_*K. pneumoniae*)

TM 1-6 of LptF and LptG form a cavity (Fig. 4a), which expands into the periplasm where the TM segments bend outwards. This cavity is 25 Å in length and 8 Å in width at its widest points (Supplementary Fig. 6). The periplasmic entrance to the cavity is surrounded by the periplasmic loops and periplasmic domains of LptF and LptG. Despite LptF and LptG sharing only 17% amino-acid sequence identity, the structures of the TMDs are strikingly similar, with an RMSD of 2.15 Å for 160-aligned Cα atoms. However, the periplasmic domain of LptG is shifted ~40 Å from that of LptF (Fig. 4b), which opens the cavity to the periplasm at the lateral gate TM5F-1G side, while the lateral gate TM5F-1G is open. The LptB_2_FG structure presented here may represent an open conformation of the lateral gate of TM5F-1G (Figs. 2c, d and 3a, b). The IM section of the internal cavity is very hydrophobic (Supplementary Fig. 7), while the section above the IM is highly positively charged (Supplementary Fig. 6c). The residues positioned inside of the cavity show a higher degree of conservation than those positioned outside of the transporter (Supplementary Figs. 7 and 8). There is an extra electron density in the cavity that could not be assigned with any confidence (Supplementary Fig. 9). We speculate that the cavity of LptB_2_FG may bind LPS (Supplementary Fig. 6c), as LPS is very hydrophobic at the lipid A and highly negatively charged near the inner core (Fig. 1a). Positively charged residues are required for LPS-specific binding in other membrane proteins^39^. The highly conserved residues K34 (TM1G) and R136 (TM3G) of LptG are found at the upper cavity of the transporter (Fig. 3b) and around this extra electron density. We hypothesized that the residues K34 and R136 may be involved in LPS extraction and transport. Functional assays indeed revealed that the double glutamic acid substitution K34E/R136E significantly affected the bacterial growth. In contrast, LptG residues, K40 and K41, are not conserved, and functional assays showed that the double-mutant K40E/K41E did not affect cell growth (Figs. 3c, d and Supplementary Fig. 7). We also expected that the internal hydrophobic cavity is important for LptB_2_FG’s function. Highly conserved hydrophobic residues F26 and L62 of LptF are located in the central cavity and around the extra electron density (Fig. 3b and Supplementary Fig. 7). We speculate that these two residues may be involved in LPS transport. A functional assay showed that the double-mutant LptF F26D/L62D was lethal (Fig. 3c, d). The two mutant LptB_2_CFG LptG K34E/R136E and LptF F26D/L62D were eluted out at the same volume as that of the wild-type LptB_2_CFG during size-exclusion chromatography (Supplementary Fig. 10), indicating that the two mutant LptB_2_CFG complexes were properly folded. These data suggest that the highly conserved residues of LptF and LptG located in the internal cavity are essential for the functionality of LptB_2_FG transporter, which is likely to be involved in interacting with the lipid A and the inner oligosaccharide core of LPS.

### Both LptF and LptG periplasmic domains are critical

The periplasmic domains of LptF and LptG adopt β-jellyroll folds, which together comprise 10 β-strands in a manner that resembles the periplasmic β-jellyroll domain of LptC (Supplementary Fig. 11). The periplasmic domains of LptF and LptG take opposite side-by-side orientations, with both LptF and LptG connecting to the hydrophobic cavity of LptB_2_FG (Fig. 2 and Supplementary Figs. 6–8).

Two-point mutations on the C-terminal strand of LptA disrupt LptA oligomerization^40^ and another mutation in the C-terminal region of LptC disrupts the LptA-LptC interaction, resulting in bacterial death^10^. To test whether the C-terminal strands of the periplasmic domains of LptF and LptG are also as important as that of LptA and LptC, we generated single proline substitutions at the C-terminal β-strands of the LptF periplasmic domain (F_D229P, F_Q231P, F_T225P, and F_R223P) and at the C-terminal β-strands of LptG periplasmic domain (G_G228P, G_W230P, G_T225P, and G_S223P) (Fig. 4b). Functional assays showed that all the mutants severely impaired the bacterial growth, except for F_T225P (Fig. 4c, d). Additionally, double proline substitutions (F_D229P/Q231P, F_T225P/R223P, G_G228P/W230P, and G_T225P/S223P) cause significant cell growth defects (Supplementary Fig. 2), suggesting these periplasmic domains are important for the function of LptF and LptG in LPS transport.

### Interactions of LptF and LptG with LptB

**Fig. 5:**
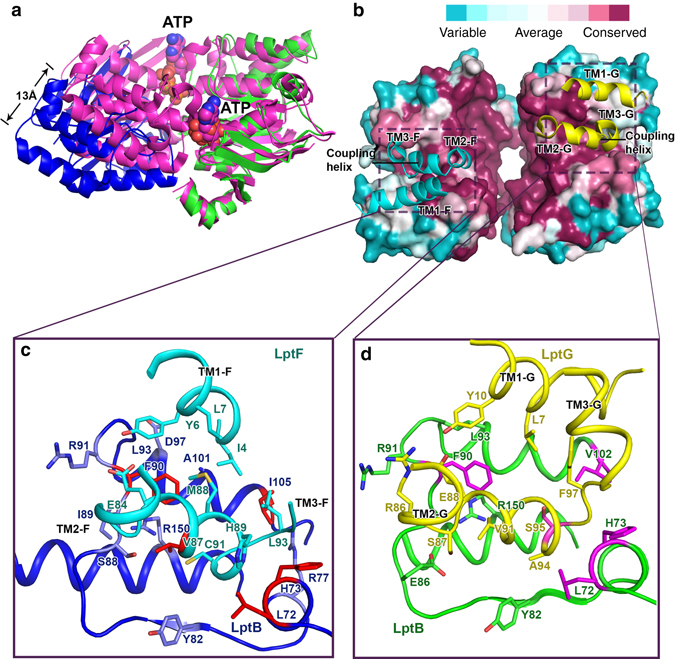
LptB structure and its interactions with LptF and LptG. **a** Superimposition of dimeric LptB of *K. pneumoniae* (*blue* and *green*) and that of *E. coli* (*magenta*) in complex with ATP (PDB access code 4QC2). The structure of *E. coli*’s dimeric LptB shifts around 13 Å from the ATP-free structure of the dimeric LptB in the *K. pneumoniae* LptB_2_FG. **b** The conserved residues of the LptB and the coupling helices of LptF and LptG. The most variable residues are in *cyan* and the most conserved residues are in *dark red*. The coupling helices of LptF and LptG, between TM2 and TM3, are located at the LptB middle groove. **c** The residues of coupling helix of LptF and TM1F (*cyan*) interact with the residues of one LptB monomer (*blue*). The residues (LptB residues L72, H73, F90, and I105, and LptF V87) that formed the UV-dependent crosslinks are shown in *red* ^41^. **d** The residues of the coupling helix of LptG and TM1G (*yellow*) interact with residues of another LptB monomer (*green*). The residues (LptB residues L72, H73, F90, and V102, and LptG S95) that formed the UV-dependent crosslinks are shown in *magenta* ^41^

The NBDs of ABC transporters hydrolyze ATP and drive the conformational changes of the TMDs to translocate their substrates across membranes through the coupling helices. The LptF-coupling helix (residues E84, V87, M88, H89, C91, and L93) interacts with LptB residues H73, R77, Y82, F90, R91, and R150, while the LptG-coupling helix (residues R86, S87, E88, V91, A94, and F97) interacts with LptB residues L72, Y82, E86, F90, R91, V102, and R150 (Fig. 5 and Supplementary Fig. 12a). Recent work confirmed LptB-F90, L72, M73, and I105 interact with coupling helices of LptF (V87) and LptG (S95) by UV-dependent crosslinking^41^. Interestingly, the LptB_2_FG crystal structure also suggests other interactions between TM1F/G and LptB through highly conserved residues (Fig. 5 and Supplementary Fig. 8).

In this structure, the aromatic side chain of LptB-F90 interacts with hydrophobic residues of LptG-(Y10 and V91) and LptF-(Y6, V87, and M88) (Fig. 5c, d). Disrupting this hydrophobic interaction by substituting the aromatic residue with alanine (LptB-F90A) was lethal, whereas an aromatic residue substitution LptB-F90Y had no impact^27^. Single mutants of other LptB residues R150A and L93F that interacted with the coupling helices of LptF (E84, V87, and M88) and LptG (E88 and V91) also affected the LPS transport^41^.

The structure of LptB in this LptB_2_FG crystal form is in a nucleotide-free state. Superimposition of the LptB protomer structure of LptB_2_FG with the *E. coli* LptB protomer structure complexed with ATP (PDB code 4QC2) reveals conformational changes within the Walker A, Q-loop, signature motif, Walker B, D-loop, and H-loop (Supplementary Fig. 12b). Superimposing the dimeric *E. coli* LptB-ATP structure with the dimeric *K. pneumoniae* LptB structure reveals a shift of 13 Å by the second LptB subunit through translocation (Fig. 5a).

## Discussion

**Fig. 6:**
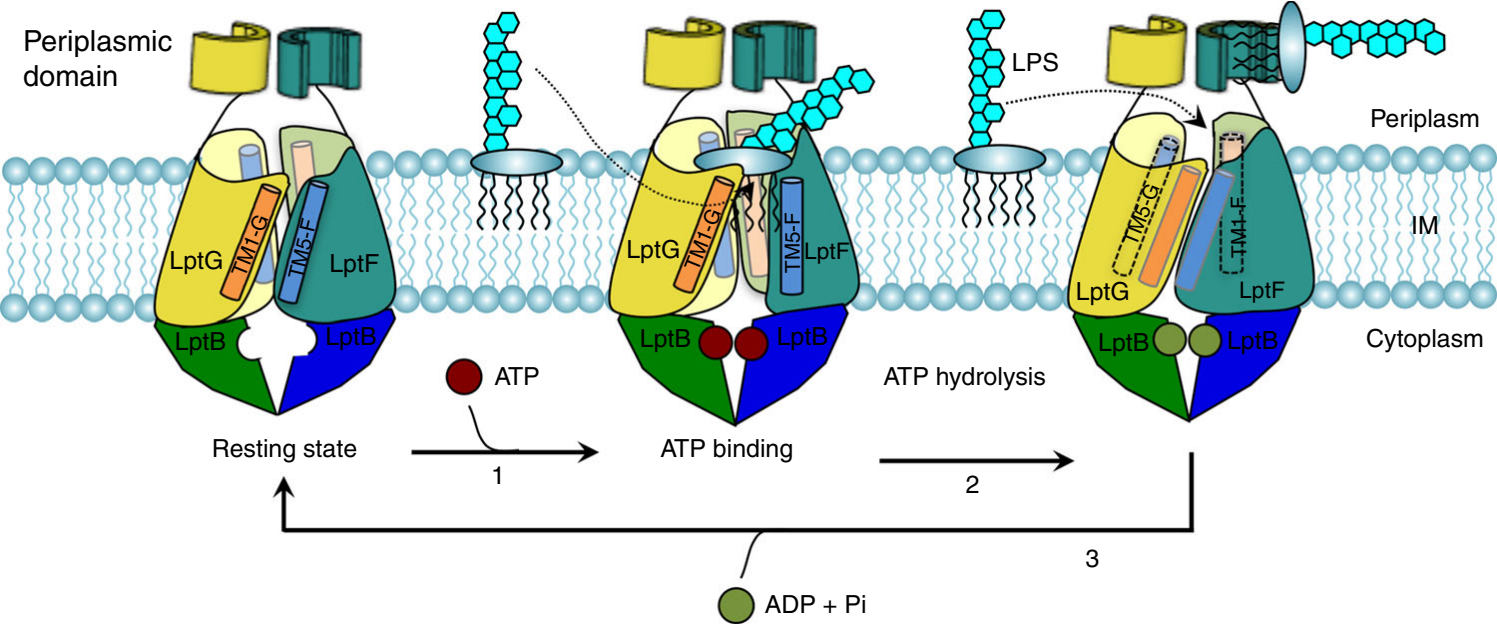
Mechanistic model for LPS transporter LptB_2_FG. LPS transporter LptB_2_FG may extract and transport LPS through a cycle of ATP-free state (resting stage), ATP-binding state, and ATP hydrolyzed state. The dimeric LptB molecules move closer to bind ATP when ATP enters into the active site of LptB (step 1). This triggers the conformational changes of TM5F-1G, which fully opens the lateral gate TM5F-1G of the transporter to extract the lipid A of LPS from the IM into the internal hydrophobic cavity (lateral opening). The lipid A is then pushed to the periplasmic domain of LptF when ATP is hydrolyzed, while the lateral gate TM5F-1G may be closed and the lateral gate TM1F-5G may be fully open to extract LPS and finally the LPS may be pushed to the periplasmic domain of LptG (step 2). The highly conserved hydrophobic residues and positively charged residues in the cavity may be involved in LPS extraction and transport. Next, ADP is released and LptB_2_FG returns to the ATP-free state (step 3) and the cycle repeats

The crystal structure of the LptB_2_FG complex uniquely represents ABC transporters like itself and LolCDE, which both possess the ability to extract large amphiphiles from the external face of the IM in advance of distinctly different periplasmic transport steps toward the OM. As an ABC exporter, this LptB_2_FG structure has large periplasmic domains. The structure register was validated by sulfur and selenomethionine anomalous data, as well as by functional assays from ourselves and other groups^41^. The extraction of LPS from the periplasmic leaflet of the IM and its transport to LptC by the LptB_2_FG complex utilizes ATP hydrolysis, as does the transport of LPS to LptA by LptC^10, 27, 28^. The crystal structure of LptB_2_FG shows that the coupling α-helices of LptF and LptG are located within a highly conserved groove of LptB (Fig. 5b). It also reveals that residues Y6, L7, I4, and of TM1F and residue Y10 of TM1G interact with residues of LptB (Fig. 5b–d). Notably, LptF and LptG’s coupling helices connect TM2F(G) and TM3F(G) within the LptB_2_FG cavity, and TM3F(G) is further connected to the periplasmic domains (Fig. 2a, b and Supplementary Fig. 12a). It has been shown previously that hydrolysis of ATP induces conformational changes in the LptB groove structure^27^. Speculatively, the binding of ATP by LptB would induce a similar conformational change to that seen in the *E. coli* form (moving the opposite protomers of LptB into close contact) and play a critical role for LptB_2_FG to extract LPS from the IM and transport LPS (Fig. 6).

The structure of multidrug ABC exporter Sav1866 complexed with ADP is in an outward-facing conformation, while the cavity surface in the IM is hydrophilic. The structure of the human sterol transporter ABCG5/ABCG8 is in an inward-facing conformation, and the structure of MsbA in complex with AMP-PNP is in an outward-facing conformation (Supplementary Fig. 13). All the previously reported ABC transporters are proposed to translocate their substrates across membranes by altering between inward- and outward-facing conformations, harnessing the energy of ATP binding and hydrolysis at their nucleotide-binding domains. The LptB_2_FG structure reveals that the LptB_2_FG cavity opens to the periplasm, while the LptB_2_FG transporter has separations between TM5F-1G, as well as TM1F-5G. We speculate that TM5F-1G and TM1F-5G may be lateral gates of LptB_2_FG transporter, whereas the lateral gate TM5F-1G is in an open form and TM1F-5G is in a closed form in this LptB_2_FG structure. In contrast to all structurally characterized ABC transporters using altering inward-facing and outward-facing conformations to translocate their substrates across membranes, the way LptB_2_FG extracts LPS is significantly different in that it extracts LPS laterally from the external leaflet of the IM and propels it along a filament that extends across the periplasm to directly deliver LPS into the external leaflet of the OM. We propose that the LptB_2_FG transporter uses alternating lateral gates (TM5F-1G and TM1F-5G) in “open” and “closed” conformations to transport LPS without it passing through the IM.

Binding and hydrolysis of ATP induce the opposing protomers of LptB to switch from the “open” to the “closed” ATP-binding states as observed when comparing the LptB structure in complex with ATP with the ATP-free LptB_2_FG structure (Fig. 5a). This conformational change would engage the coupling helices of the TMDs to open and close LptBFG’s lateral gates TM5F-1G or TM1F-5G at either side of the LptB_2_FG transporter (Figs. 2 and 6). This would allow the lateral entry of LPS from the IM into the complex when ATP is bound from either TM5F-1G or TM1F-5G; The lipid A of LPS would be loaded into the highly hydrophobic cavity from the periplasmic side of the IM with the help of the highly positively charged residues at the upper cavity and be driven to the periplasmic domain of either LptF or LptG (Fig. 6). Release of ADP from LptB would induce conformational changes to close the lateral gate (TM5F-1G or TM1F-5G). The periplasmic β-jellyroll domains of LptF and LptG are similar to LptC and LptA (Supplementary Fig. 11), and the crystallographic packing reveals a β-jellyroll extension between neighboring LptF subunits (Supplementary Fig. 4), indicating that the periplasmic domain may interact in a similar manner with LptC for LPS transport. The LptF R212G/S mutants on the periplasmic domain can suppress the lethality of LptC-depleted strain, suggesting that the periplasmic domain of LptF plays a critical role for forming the LPS transport protein complex and transporting LPS^42^.

Both periplasmic domains of LptF and LptG may be involved in transporting LPS as almost all single or double proline substitutions of the periplasmic domains of LptF and LptG kill bacteria (Fig. 4), suggesting that either lateral gate TM5F-1G or TM5G-1F of LptB_2_FG may extract LPS from the periplasmic leaflet of the IM. The LptB_2_FG structure presented here may represent only one of the transporter’s conformational states, where it takes LPS from the TM5F-1G opening to the cavity and then delivers LPS to the periplasmic domain of LptF. We speculate that LptB_2_FG may adopt another conformation, which allows the transporter to obtain LPS similarly, but from the lateral gate TM1F-5G to deliver LPS to the periplasmic domain of LptG.

The LptB_2_FG electron density map contains an unassigned density within the central hydrophobic cavity. Functional assays showed that double-mutants LptG_K34E/R136E and F_F26D/L62D within the cavity result in severe cell growth defects, suggesting that this complex may extract LPS from the IM to the central cavity. This is consistent with a report that wild-type LptB_2_FG of *Burkholderia cenocepacia* is unable to export LPS lacking 4-amino-4-deoxy-C-arabinose modification with the lipid A or core oligosaccharide, while the LptG D31H mutant can export the unmodified LPS^43^. Bioinformatics analysis suggests that residue LptG_D31 is structurally equivalent to LptG_K34 of *K. pneumoniae*, occupying the same position in the cavity. Additionally, LptC and LptA could not extract LPS directly from the IM^28^. Taken together, these data suggest that the ABC transporter LptB_2_FG extracts LPS from the IM and helps to transport it to the OM. The LptB_2_FG structure now reveals that the periplasmic LptCAD β-jellyroll track is likely continuous with the LptFG β-jellyroll motifs that interface between LptCAD and the LPS lipid A moieties buried in the external leaflet of the IM. A continuous hydrophobic tunnel is thus expected to connect the internal leaflet of the OM with the external leaflet of the IM. LptB_2_-catalyzed ATP hydrolysis is expected to drive LPS transport by staging unique conformational changes felt at the interface, where LPS molecules are disposed orthogonally between the β-jellyroll track and the external leaflet of the IM. The mechanics of LPS transport is thus restricted within the external leaflet and periplasmic domains of the LptB_2_FG complex, with the impulse coming from the cytoplasm in the absence of any associated transmembrane lipid flipping events.

In summary, our structural and functional studies suggest that LptB_2_FG uses an alternating lateral access mechanism for LPS extraction from the IM external leaflet and into an interior LPS-binding cavity. LPS is then extruded orthogonally into the LptCAD filament, which forms a continuous hydrophobic groove capable of directly interconnecting the lipophilic domains of two distinct membrane systems. Transport is energized by ATP hydrolysis in the cytoplasm, but the lipid substrate is not itself flipped across the membrane; instead, it is laterally extracted and orthogonally ejected.

While this manuscript was under review, the LptB_2_FG structure of *Pseudomonas aeruginosa* was published^44^. The LptB_2_FG structure of *P. aeruginosa* is at a different conformation to that of LptB_2_FG of *K. pneumoniae* (Supplementary Fig. 14), where the periplasmic domains of the LptB_2_FG structure of *P. aeruginosa* rotate around 90° to the lateral gate TM5F-1G, generating a periplasmic opening for the lateral gate TM1F-5G. The lateral gate TM1F-5G of the *P. aeruginosa* LptB_2_FG is in an open conformation, while the lateral gate TM5F-1G is in a closed conformation (Supplementary Fig. 14 and Supplementary Movies 1 and 2). This structure suggests that the transporter may extract LPS from the lateral gate TM1F-5G to the internal cavity and transport to the periplasmic domain of LptG.

## Methods

### Protein expression and purification of LptB_2_FG complex

The gene fragments containing LptB and LptF-LptG of *K. pneumoniae* were amplified by PCR individually. The two fragments were subsequently ligated into the pTRC99a plasmid with *Eco*RI/*Kpn*I restriction digestion for LptB and *Kpn*I/*Xba*I for LptF-LptG, respectively. The recombinant plasmid including an octa-histidine (8 × His) at the C terminus of the *LptB* was transformed into *E. coli* *C43 (DE3)* strain (Novagen) for protein expression. The bacterial cells were grown in Luria broth (LB) supplemented with antibiotic (ampicillin 100 µg ml^−1^) at 37 °C until the optical density of the culture reached 0.6 at a wavelength of 600 nm (OD_600_). Then, LptB_2_FG co-expression was induced with 0.1 mM isopropyl β-D-thiogalactopyranoside (IPTG) at 20 °C for 16 h.

Cells were harvested by centrifugation, and re-suspended in 20 mM Tris-Cl, pH 7.8, and 150 mM NaCl supplemented with cOmplete protease inhibitor tablets (Roche), 1 µg ml^−1^ DNase (Sigma-Aldrich) and 1 mM phenylmethylsulphonyl fluoride (PMSF, Sigma-Aldrich). The cells were broken twice broken using a cell disrupter at 30,000 psi (Constant Systems Ltd). Cell debris was removed by ultra-centrifugation at 18,000×*g* for 15 min at 4 °C. The cell membrane fraction was collected with ultra-centrifugation at 100,000×*g* for 1 h at 4 °C, and was solubilized with 20 mM Tris-Cl, pH 7.8, and 300 mM NaCl, 10 mM imidazole and 1% (w/v) n-dodecyl-β-D-maltopyranoside (DDM) (Anatrace) supplemented with cOmplete protease inhibitor tablets (Roche) at room temperature for 20 min. The suspension was then ultra-centrifuged at 100,000×*g* for 30 min before being loaded onto a 5 ml HisTrap HP column (GE Healthcare) and washed with 20 mM Tris-Cl, pH 7.8, 300 mM NaCl, 60 mM imidazole, 0.6% (w/v) 5-cyclohexyl-1-pentyl-β-d-maltoside (Cymal-5) (Anatrace), 0.03% (w/v) DDM and 0.04% decyl maltose neopentyl glycol (DMNG). The LptB_2_FG complex was eluted with 20 mM Tris-Cl, pH 7.8, 300 mM NaCl, 300 mM imidazole, 0.6% (w/v) Cymal-5, 0.03% DDM, and 0.04% DMNG. The eluted LptB_2_FG complex was incubated with 3U lysine endoproteinase C (Sigma) for 15 min, and was further purified using a HiLoad 16/600 Superdex 200 prep grade column (GE Healthcare) in 20 mM Tris-Cl, pH 7.8, 150 mM NaCl, 0.6% (w/v) Cymal-5, 0.03% DDM, and 0.04% DMNG. Protein fractions with the highest purity of LptB_2_FG were collected and concentrated to 10–15 mg ml^−1^. The SeMet incorporated LptB_2_FG proteins from *S. flexneri* were overexpressed in M9 medium plus the nutrition mix (molecular dimensions) with amino acids mix inhibition and 0.1 mM IPTG for 20 h at 20˚C temperature. The SeMet LptB_2_FG was purified using the same method as the native LptB_2_FG described as the above.

The *E. coli* wild-type LptB_2_CFG and the mutants of LptF F26D/L62D and LptG K34E/R136E were expressed and purified using the same protocol as that of the LptB_2_FG of *K. pneumoniae*, and purified by size-exclusion chromatography using a column HiLoad 16/600 Superdex 200 pg column in 20 mM Tris-Cl, pH 7.8, 150 mM NaCl, 5% glycerol, and 0.05% DDM. The chromatograms of wild-type LptB_2_CFG, mutants LptF F26D/L62D and LptG K34E/R136E were integrated and compared (Supplementary Fig. 10).

### Crystallization and data collection

The LptB_2_FG crystallization trials were performed using 1 µl of protein and 1 µl of reservoir solution and the sitting-drop vapor diffusion technique at room temperature. The best crystals of LptB_2_FG were obtained in 0.1 mM MES pH 6.5, 0.1 M sodium chloride, 0.1 M lithium sulfate, and 24% PEG 300 within 8 days. Crystals were harvested after 3–4 weeks and cryoprotected by supplementing the crystallization solution with 20% glycerol, before being flash frozen in liquid nitrogen. Platinum derivatives were obtained by soaking the crystals for 4 h in the crystallization solution with 2 mg ml^−1^ potassium tetranitroplatinate (II) K_2_Pt(NO_2_)_4_. The data sets were collected at beamline I03, Diamond Light Source, UK, at the platinum L3 edge. The data were processed using XIA2^45^ with DIALS^46^ and scaled using AIMLESS^47^. Anisotropic diffraction was observed and correction was applied during data processing. Crystals belonged to space group *I*2_1_2_1_2_1_ with unit-cell dimensions: *a* = 105.5 Å, *b* = 210.8 Å, *c* = 258.9 Å, and *α* = *β* = *γ* = 90°. The SeMet incorporated LptB_2_FG crystals from *S. flexneri* were obtained in 0.2 M sodium acetate trihydrate, 0.1 M MES pH 6.5 and 28% v/v PEG 400 for 21 days. The wavelength 0.9795 Å was used for SeMet crystals at beamline I03. The SeMet crystals belonged to space group *P*2_1_2_1_2_1_ with the cell dimensions *a* = 110.15 Å, *b* = 124.53 Å, *c* = 398.09 Å, and *α* = *β* = *γ* = 90° (see “Methods” section and Table 1).

The LptB_2_FG sulfur anomalous data were collected at 1.7712 Å and the data were processed using XIA2^45^ with DIALS^46^ and scaled using AIMLESS^47^. LptB_2_FG co-crystallization with nucleotide (ATP, ADP, AMP, and AMP-PNP) was attempted; however, no nucleotide-binding structure was obtained.

### Structure determination and model building

The platinum positions in the Pt-derived crystals of the *I*2_1_2_1_2_1_ space group were determined by the SAD method using SHELX suite^48^ and were successful in locating platinum sites but did not result in a readily interpretable map. Data from six sweeps across three isomorphous crystals were combined to increase the anomalous multiplicity to around 60-fold with a diffraction limit of 3.7 Å and this produced a map where the 12 transmembrane helices were clearly visible along with the electron density for the LptB domains. Anisotropy correction of the raw data set was performed using the STARANISO web server (http://staraniso.globalphasing.org/cgi-bin/staraniso.cgi) with a surface threshold of 1.2I/σ(*I*) and approximate vector 0.1 a* + 0.6 b* + 0.8 c*.

The phasing and density modification of this anisotropy corrected data were performed with PHENIX^49^ resulting in a significantly more interpretable map with the main chain of a single copy of the complex clearly visible along with some side-chain density, particularly around the transmembrane region. Four-ordered platinum sites were found with an overall figure of merit of 0.33. The very high solvent content of these crystals (77%, *V*_m_ = 5.4 Å^3^ Da^−1^) aided density modification greatly. This initial density modified map is shown along with the final refined model and anomalous Fourier density in Supplementary Fig. 3b. The best data we collected can reach to 3.3 Å, but the completeness of the high-resolution shell was dropped to 10% after the anisotropy correction. We used this data for the initial structure building, while we performed the final refinements using the 3.7 Å data. The best electron density map with the refined structure is shown in Supplementary Fig. 5.

Two molecules of high-resolution models of LptB (PDB code 4QC2) were placed into the density at the base of the transmembrane region and adjusted to fit by using COOT^50^. The transmembrane regions of LptF and LptG were relatively straightforward to build using standard helices and registry was assigned using the sulfur anomalous data. The peaks in the anomalous Fourier map of the sulfur were generated using PHENIX^49^. The initial registry assignment of the structure is performed using the peaks in the sulfur Fourier maps with 10 peaks at 3 sigma (Supplementary Fig. 3a). The side chains of the LptB_2_FG structure is further registered using the SeMet sites identified from the anomalous map of *S. flexneri*, as the amino-acid sequences of LptB_2_FG from *S. flexneri* share conserved methionine residues with that of the *K. pneumoniae* (Supplementary Figs. 3c, 15–17). In addition, the platinum sites are found near methionine, arginine, and histidine residues as would be expected for this metal. Model refinement was performed using BUSTER^51^ with each chain assigned amino acids a rigid body for TLS. NCS was used to restrain the two LptB domains. We used the 3.7 Å data for the final refinement. The final model has *R*_work_ and *R*_free_ of 0.29/0.32, respectively, and further statistics are given in Table 1.

### Site-directed mutagenesis and functional assays

All single or double mutations were generated following the site-directed mutagenesis protocol of Liu and Naismith^52^. The mutations were amplified by PCR using Q5^®^ hot start high-fidelity DNA polymerase. To avoid ampicillin already used to select the *E. coli* lptFG deletion strain NR1113, the pTRC99a plasmid’s ampicillin resistance gene was replaced by a kanamycin-resistance gene. Named as pTRC99a-Kan, this plasmid was used as the template for the LptBFG mutagenesis, which included an octa-histidine tag (8 × His) at the C terminus of the LptB.

The construct had a Flag tag inserted at residue 230 of LptF (LptF-230-Flag), a c-Myc tag at residue 228 of LptG (LptG-228-Myc) or a Flag tag at residue 138 of LptF (LptF-138-Flag), and a c-Myc tag at 144 of LptG (LptG-144-Myc) to generate two plasmids, which we named as LptBF230G228 and LptBF138G144, respectively. All mutations have been confirmed by DNA sequencing.

Primers for generating the LptF and LptG tags are listed as below:

K12_F230_Flag_F: GATTACAAAGATGACGACGATAAA CAGGCGATCATTGGTCACCAGGC

K12_F230_Flag_R: TTTATCGTCGTCATCTTTGTAATC ATAATCCTGGAAGTCCGTAATGCGGAAATCACG

K12_G228_Myc_F: GAACAAAAACTCATCTCAGAAGAGGATCTG ACCTGGAAAACCAACCTCACGCC

K12_G228_Myc_R: CCTCTTCTGAGATGAGTTTTTGTTC GCCGCTCACCGTCTGCGAAC

K12_F138_Flag_F: GATTACAAAGATGACGACGATAAA CCTGGCATGGCGGCGCTG

K12_F138_Flag_R: TTTATCGTCGTCATCTTTGTAATC GTTCGCTTTCGCTTCTGCTAACACTTCATCCTG

K12_G144_Myc_F: GAACAAAAACTCATCTCAGAAGAGGATCTG TTGCTCTCTACCCAGCAAGGCTTATGG

K12_G144_Myc_R: CCTCTTCTGAGATGAGTTTTTGTTC CGAGCCGCCGTACATCGCC

These single or double mutants were transformed into the *E. coli* lptFG-depleted NR1113 strain^8^ respectively. The transformed *E. coli* cells were grown on LB agar plate supplemented with antibiotics (kanamycin 50 µg ml^−1^) and 0.2% L-arabinose at 37 °C for 12 h. Single colonies of each transformation were inoculated into 5 ml LB medium supplemented with above antibiotics and 0.2% (w/v) L-arabinose. The cells were cultured in an incubator at 200 rpm and at 37 °C for 12 h. Subcultured cells were used for the functional assays. The *E. coli* NR1113 with the empty plasmid pTRC99a-Kan was used as the negative control, while the NR1113 strain with the plasmid pTRC99a-Kan-LptBF(Flag)G(Myc) or the NR1113 strain in 0.2% L-arabinose was used as the positive control. For functional assays, the cells were harvested and washed twice and then diluted in sterile LB medium to OD_600_ nm of 0.5. Ten-fold serial dilution functional assays were performed. The dilution range was from 10^−1^ to 10^−6^, and 5 µl of the diluted cells was dripped onto the LB agar plates containing kanamycin 50 µg ml^−1^. Cell growth was observed after overnight culture at 37 °C. All the assays have been performed in triplicate.

### Western blot

The protein expression levels of LptF and LptG were determined by western blotting. An aliquot of 0.5 ml of overnight cultures of transformed NR1113 cells with wild type or LptB_2_FG mutants was inoculated into 50 ml LB supplemented with antibiotics (kanamycin 50 µg ml^−1^) and 0.2% L-arabinose. The cells were cultured at 37 °C for 6 h and harvested by centrifugation. The cells were re-suspended in 1 ml buffer containing 20 mM Tris-Cl, pH 7.8, and 150 mM NaCl supplemented with cOmplete protease inhibitor tablets (Roche), 1 µg ml^−1^ DNase (Sigma-Aldrich) and 1 mM PMSF. The cells were lysed by sonication for 45 s on ice. The membrane fraction was harvested and solubilized with 2% DDM for 20 min at room temperature. The un-dissolved debris was removed by centrifugation at 13,000×*g* for 10 min at 4 °C. The supernatant was loaded to a Ni^2+^-NTA column and washed with a buffer containing 0.05% DDM, 20 mM Tris-Cl pH 7.8, 150 mM NaCl, and 30 mM imidazole. The protein was eluted with 0.05% DDM, 20 mM Tris-Cl pH 7.8, 150 mM NaCl, and 500 mM imidazole. The eluted samples were mixed with 4× SDS–PAGE loading buffer and incubated at 98 °C for 10 min. The samples were centrifuged at 13,000×*g* for 1 min, and 10 µl of each sample was loaded onto 4–12% Bis-Tris Plus SDS–PAGE Gel (Invitrogen) for the immunoblot analysis.

The proteins were transferred to the PVDF membrane using the Trans-Blot Turbo Transfer Starter System (Bio-Rad) at 20 V for 20 min. The PVDF membranes were blocked in 10 ml of protein-free T20 (TBS) blocking buffer (Fisher) at 4 °C for 1 h. The membranes were incubated with 10 ml of anti-Flag (Sigma, Catalog No: F3165) or anti-Myc monoclonal antibody (1:300 dilution) (Sigma, Catalog No: A5963) at room temperature for 1 h. The membranes were washed with PBST four times and then incubated with IRDye 800CW goat anti-mouse IgG (1:20,000 dilution) (LI-COR) for 30 min. The membranes were washed with PBST four times and PBS two times. The images were acquired using the LI-COR Odyssey (LI-COR).

### ATPase activity assay

ATPase activity was performed using ATPase/GTPase Activity Assay Kit (Sigma). *E. coli* C43 (DE3) cells harboring lptBFG (or lptBFG mutant) plasmid or LolCDE plasmid were cultured in 1 L LB medium. The protein overexpression and the cell collection were performed using the same protocol as described in the protein purification and expression method section. Cells were disrupted twice by passing a cell disruptor at 30,000 psi. The membrane fraction was harvested by the ultra-centrifugation at 100,000×*g* for 30 min and solubilized in 1% DDM followed by another ultra-centrifugation. The supernatants of each sample were loaded onto a gravity column containing pre-balanced 200 µl of Ni^2+^-NTA beads. The LptB_2_FG or LolCDE complex was washed with 15 column volumes of wash buffer (50 mM imidazole 20 mM Tris-Cl pH 8.0, 300 mM NaCl, and 0.05% DDM), and eluted using the elution buffer (250 mM imidazole, 20 mM Tris-Cl pH 8.0, 300 mM NaCl, and 0.05% DDM).

The protein concentration of all samples was determined with detergent compatible Pierce BCA Protein Assay Kit (Thermo Scientific) according to the manufacture’s instruction. Briefly, 2.5 µl of purified protein was diluted to 25 µl for the BCA assay. The albumin (BSA) was used as the standards. An aliquot of 200 µl of working reagent (made by mixing reagent A and reagent B at 50:1 volume ratio) was added to each sample and incubation at 37 °C for 30 min. The absorbance at the 562 nm was measured and the protein concentration of each sample was determined.

The ATPase activity assay was performed in 96-well plates. An aliquot of 1 µl of each sample was mixed with 4 µl 0.5% DDM TBS and 5 µl assay buffer (ATPase/GTPase Activity Assay Kit) to make 10 µl of ATPase activity assay sample. The phosphate standards and blank control for colorimetric detection was prepared according to the manufacturer’s instructions of ATPase/GTPase Activity Assay Kit (Sigma). An aliquot of 30 µl reaction mix (made by 20 µl assay buffer plus 10 µl 4 mM ATP solution) was added into each ATPase activity assay sample. After incubation at room temperature for 15 min, 200 µl reagent (ATPase/GTPase Activity Assay Kit) was added into each sample to terminate the reaction and all samples were incubated for additional 30 min. The absorbance at 600 nm was measured. All assays were repeated six times. ATPase activities of all samples were determined using the mean value of the samples according to the linear regression of standards.

### Data availability

The atomic coordinates and structure factors of LptB_2_FG are deposited at the Protein Data Bank under access code 5L75. Other data are available from the corresponding authors on reasonable request.

## Electronic supplementary material

Supplementary Information

Supplementary Movie 1

Supplementary Movie 2
