# Supplementary material for: Structural and functional insights into the lipopolysaccharide ABC transporter LptB2FG
Source: Nat Commun. 2017 Aug 9;8:222. doi: 10.1038/s41467-017-00273-5 (PMC5548808; doi:10.1038/s41467-017-00273-5)
Supplement: Supplementary file 1 — Supplementary Information [file 41467_2017_273_MOESM1_ESM.pdf]

File name: Supplementary Information
Description: Supplementary figures and supplementary tables.

File name: Supplementary Movie 1
Description: The conformational changes from the $LptB_2FG$ structure of *K. pneumonia* to that of the $LptB_2FG$ of *P. aeruginosa*. The side view of the lateral gate TM1F--TM5G. The periplasmic domains rotate around 90°, while the conformation of this lateral gate TM1F--TM5G is from the closed state ($LptB_2FG$ of *K. pneumonia*) to the open state ($LptB_2FG$ of *P. aeruginosa*).

File name: Supplementary Movie 2
Description: The conformational changes from the $LptB_2FG$ structure of *K. pneumonia* to that of the $LptB_2FG$ of *P. aeruginosa*. The side view of the lateral gate TM5F--TM1G. The periplasmic domains rotate toward to this lateral gate TM5F--TM1G, while the conformation of this lateral gate TM5F-TM1G is from the open state ($LptB_2FG$ of *K. pneumonia*) to the closed state ($LptB_2FG$ of *P. aeruginosa*).

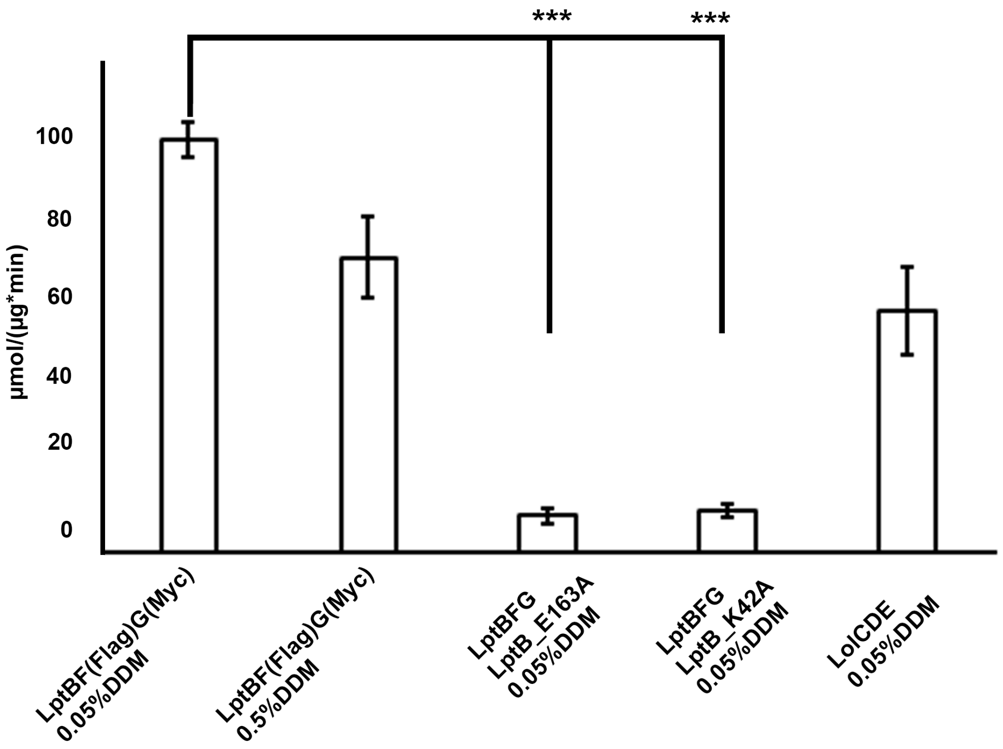

Supplementary Figure 1. Purified $LptB_2FG$ has ATPase Activity. Purified *E. coli* LptBF(Flag)G(Myc) transporter has ATPase activity in different concentrations of detergent DDM. The *E. coli* $LptB_2FG$ protein complex with LptB_E163A or LptB_K42A mutation shows significant lower ATPase activity than that of LptBF(Flag)G(Myc). *E. coli* lipoprotein transporter LolCDE is included in the ATPase activity assay as a system control. The detergent DDM has a minor affection on the ATPase activity of $LptB_2FG$. The data was analysed using one-way ANOVA, n=3; $P<0.01$; graphs show mean±S.E.M.

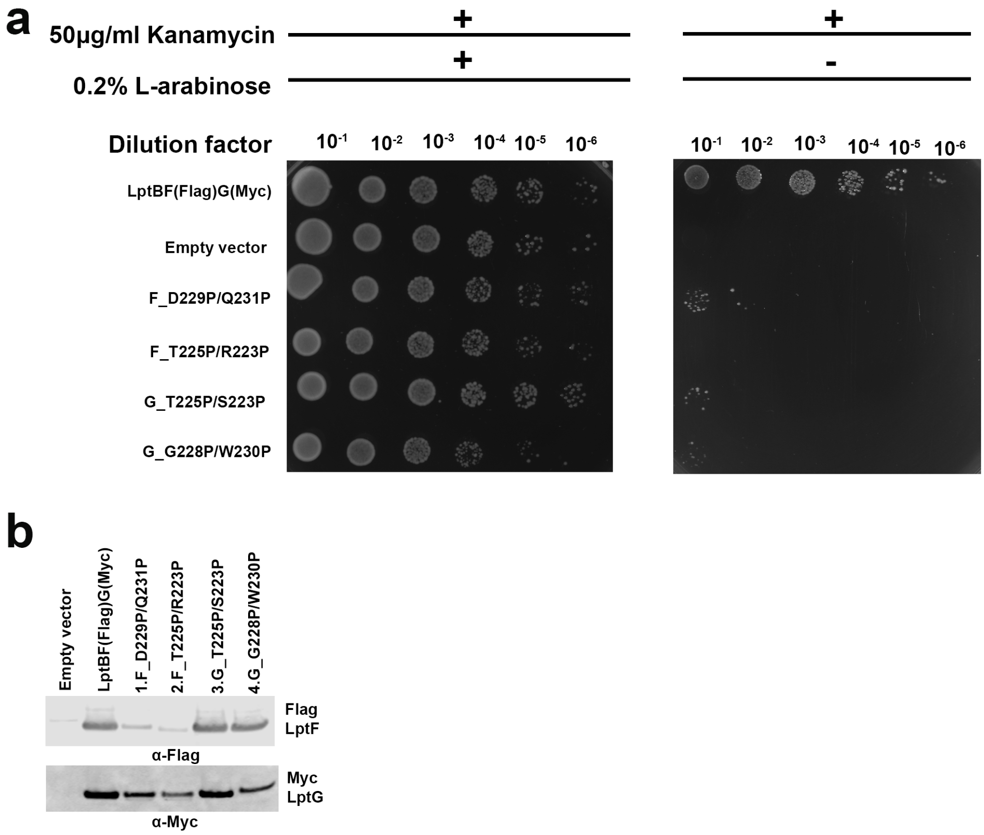

Supplementary Figure 2. Complementation assays of NR1113 with wild type and mutant $LptB_2FG$ complexes. The NR113 depletion strain expresses *lptFG* genes from an L-arabinose inducible promoter. a. Functional assays of the double proline mutants. NR1113 cells were transformed with empty vector (pTRC99a_Kan) or the vector encoding LptBF(Flag)G(Myc) as negative and positive controls, respectively, or with LptBF(Flag)G(Myc) double proline mutants. All bacterial cultures were adjusted to an OD600nm =0.5 with fresh medium, serially diluted 1:10 as indicated on the top of the figure and then replica plated in agar plates with (+) or without (-) 0.2% L-arabinose. b. Detection of protein expression levels of LptF(Flag)G(Myc) of the positive control, negative control, and the double proline mutants by Western blotting. The bacteria cells for Western blotting were cultured in the presence of 0.2% L-arabinose.

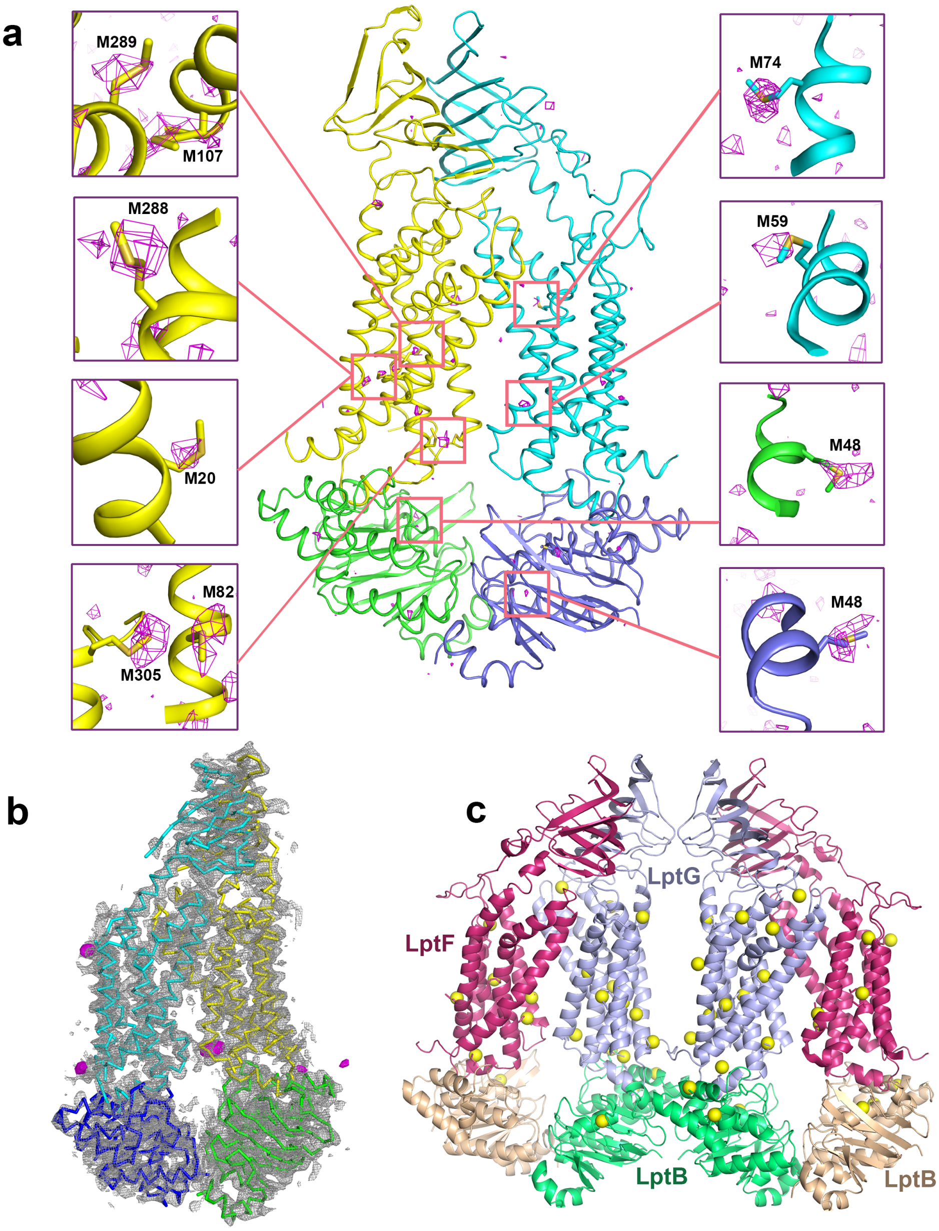

Supplementary Figure 3. LptB$_2$FG complex structure of *K. pneumoniae* was registered using sulfur data and *S. flexneri* Selenomethionine data. a. The anomalous difference Fourier map of sulfur contoured at 3σ is shown in pink. The map of sulphur was used for the model building. b. The final refined structure of LptB$_2$FG is shown as a Cα trace superimposed on the initial density modified experimental map (grey) contoured at 1.2σ. The anomalous difference Fourier map contoured at 7σ is shown in pink, highlighting the location of the Pt sites. Both maps were calculated from data that was corrected for anisotropy along the surface defined by $I/\sigma I<1.2$. c. The data from *S. flexneri* LptB$_2$FG selenomethionine (Se-Met) incorporated crystal are

collected and the anomalous signal map is generated for amino acids sequence registry. The yellow spheres display the Se-Met sites identified by phaserEP from the Se-Met incorporated crystal dataset per asymmetric unit. The Se-Met incorporated crystal belongs to space group *P212121*. There are two transporters per asymmetric unit. LptF and LptG are shown in raspberry and slate respectively. The two copies of LptB are shown in lime green or light orange.

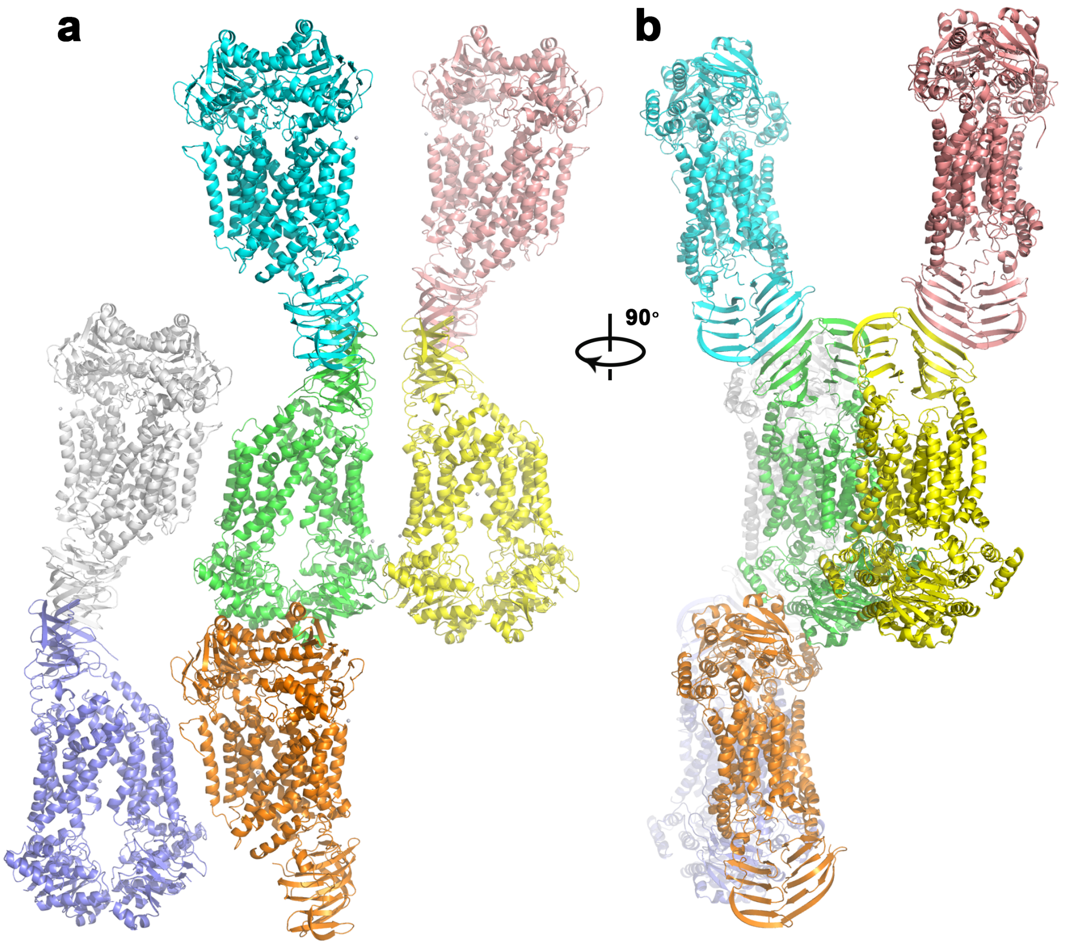

Supplementary Figure 4. Crystal packing of $LptB_2FG$. a. The $LptB_2FG$ molecules are packed in the crystals via interactions of the neighbouring LptF periplasmic domains, neighbouring LptB molecules, and neighbouring LptG TM domains. The transporters are in cyan, raspberry, grey, green, yellow, slate and orange. b. Rotation of 90° of Figure A along Y-axis. The neighbouring periplasmic domains of LptF stack against each other.

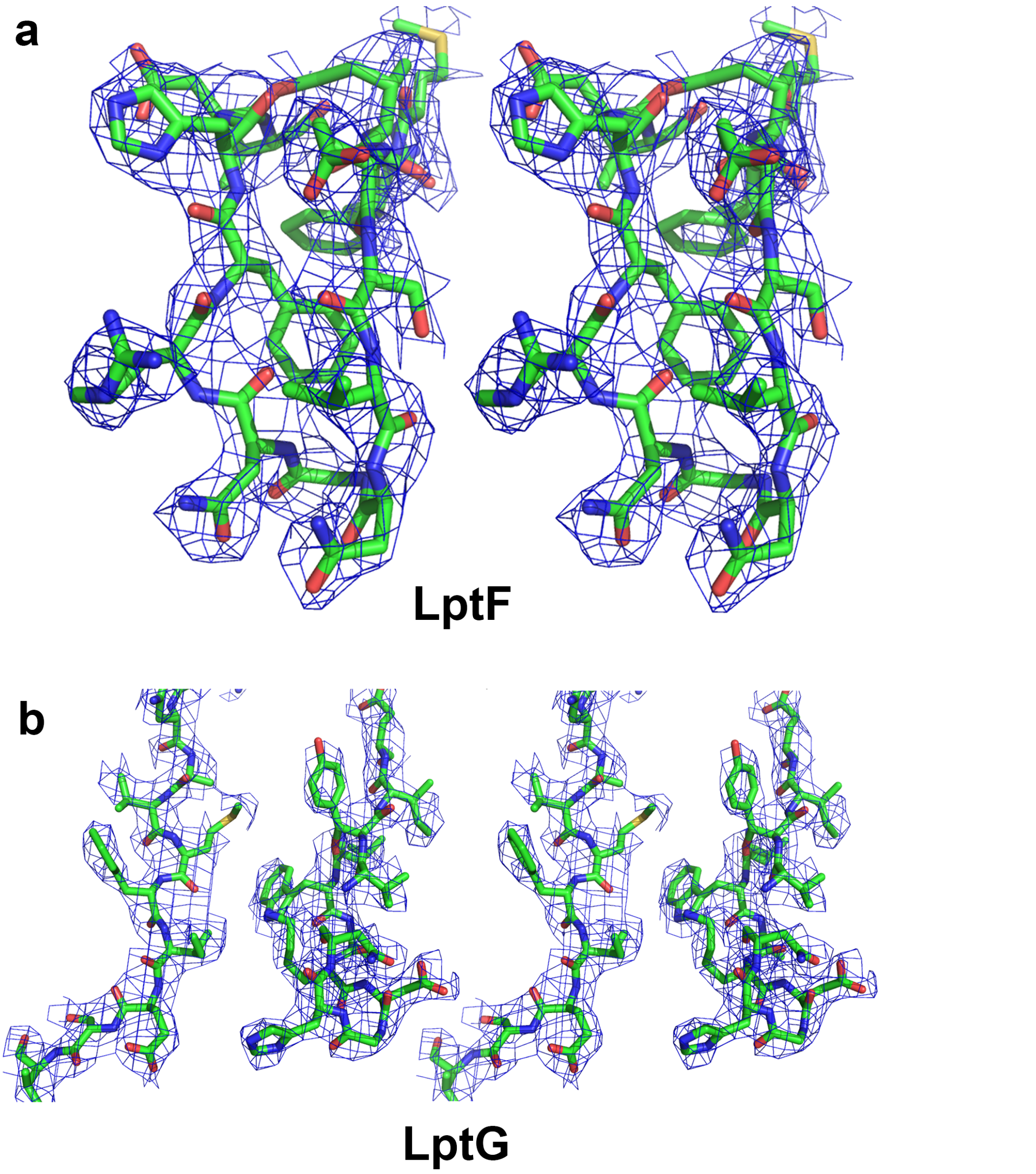

Supplementary Figure 5. The stereo electron density map of parts of $LptB_2FG$. The 2Fo-Fc electron density map is shown, contoured at 1σ. a. a stereo electron density map of a part of LptF. b. a stereo electron density map of a part of LptG.

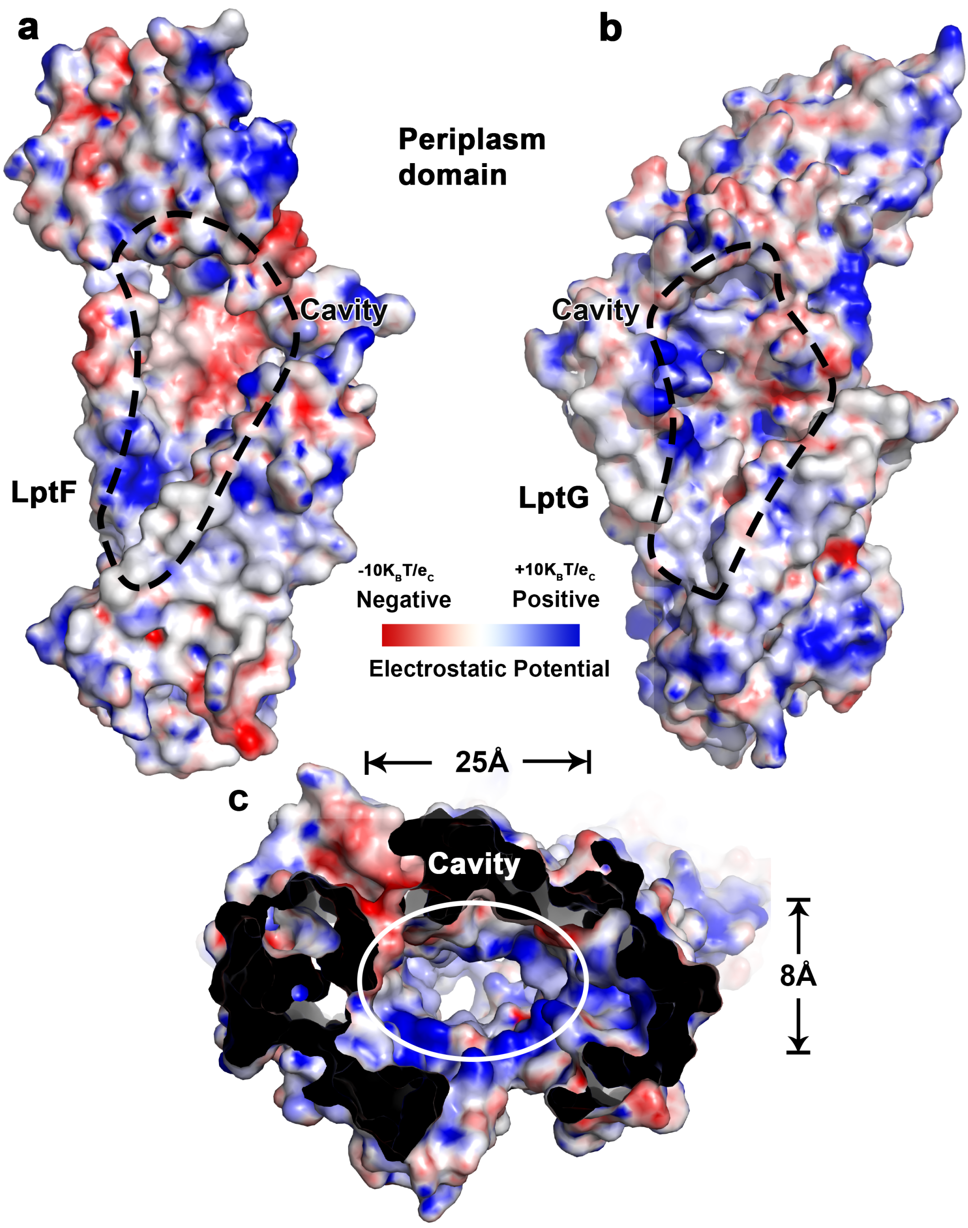

Supplementary Figure 6. Electrostatic potential map of LptF, LptG and $LptB_2FG$. The cavity of $LptB_2FG$, shown in the dotted circular lines (black). The cavity in the membrane section is very hydrophobic, while the cavity in the periplasm is highly positively charged. This feature may be important for LPS binding, as the lipid A of LPS is hydrophobic, while the core oligosaccharide is highly negatively charged. a. The electrostatic potential map of LptF. b. The electrostatic potential map of LptG. c. The electrostatic potential map of the cavity of $LptB_2FG$**.** The cavity is about 25 Å in length and 8 Å in width. The cavity is shown in the circular line (white). The figures were colored by electrostatic charge from red ($-10K_BT/e_C$, in which K_B is the Boltzmann Constant, T is temperature and e_C is the electron charge) to blue ($+10\ K_BT/e_C$).

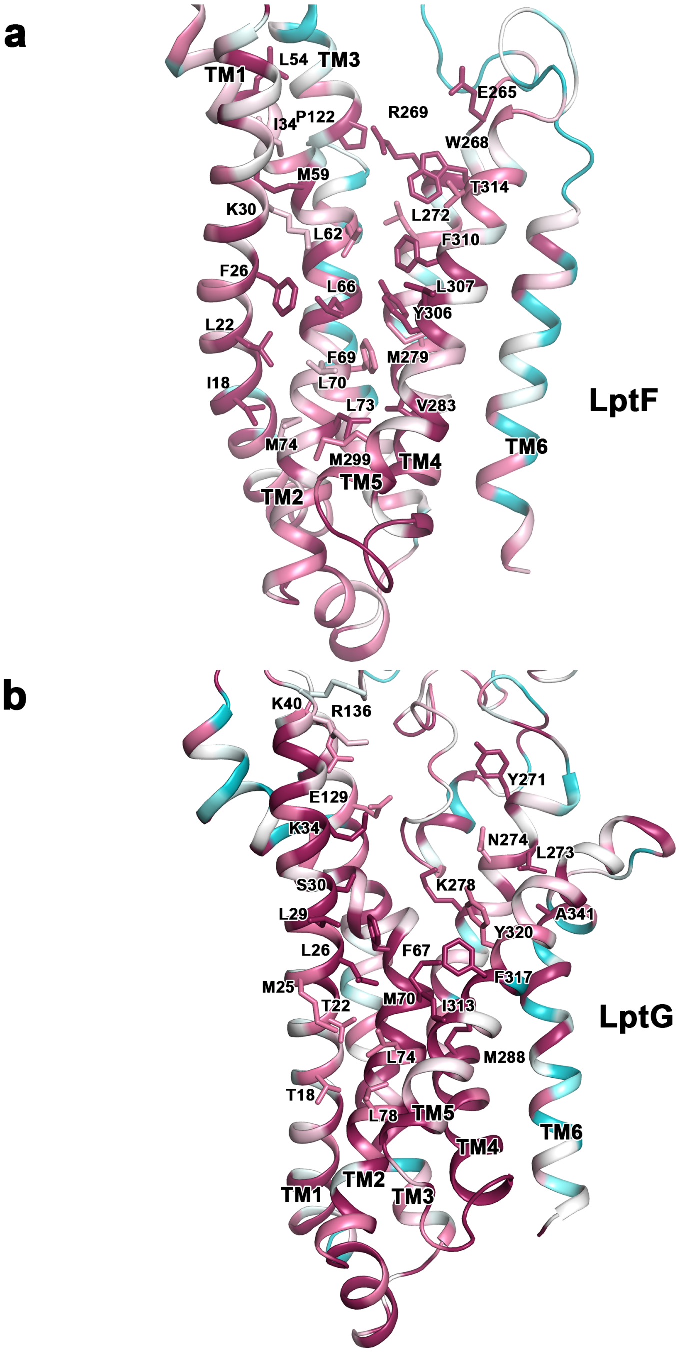

Supplementary Figure 7. The highly conserved residues in the cavity. The conserved residues of $LptB_2FG$ were analysed using the Consurf server. The conserved residues were identified from 150 homologues of LptF and LptG. The most variable residues are in cyan, and the most conserved residues are in dark red. The highly conserved residues of a. LptF; b. LptG.

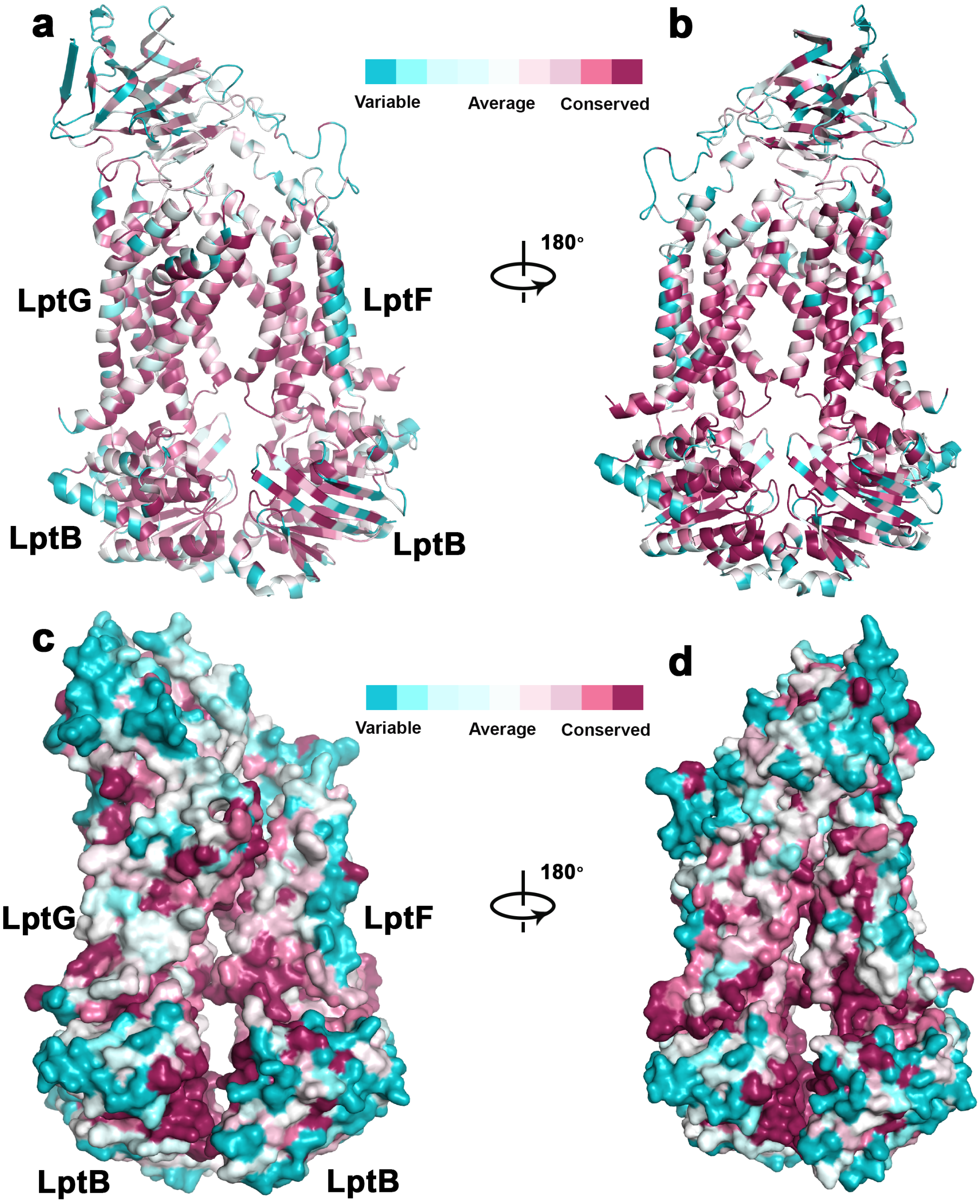

Supplementary Figure 8. Conserved residues of the $LptB_2FG$. The conserved residues of $LptB_2FG$ were analysed using the Consurf server. The conserved residues were identified from 150 homologues of LptF and LptG and 52 homologues of LptB. The most variable residues are in cyan, and the most conserved residues are in dark red. The $LptB_2FG$ transporter is shown as a cartoon (a and b) and as a surface (c and d) renderings.

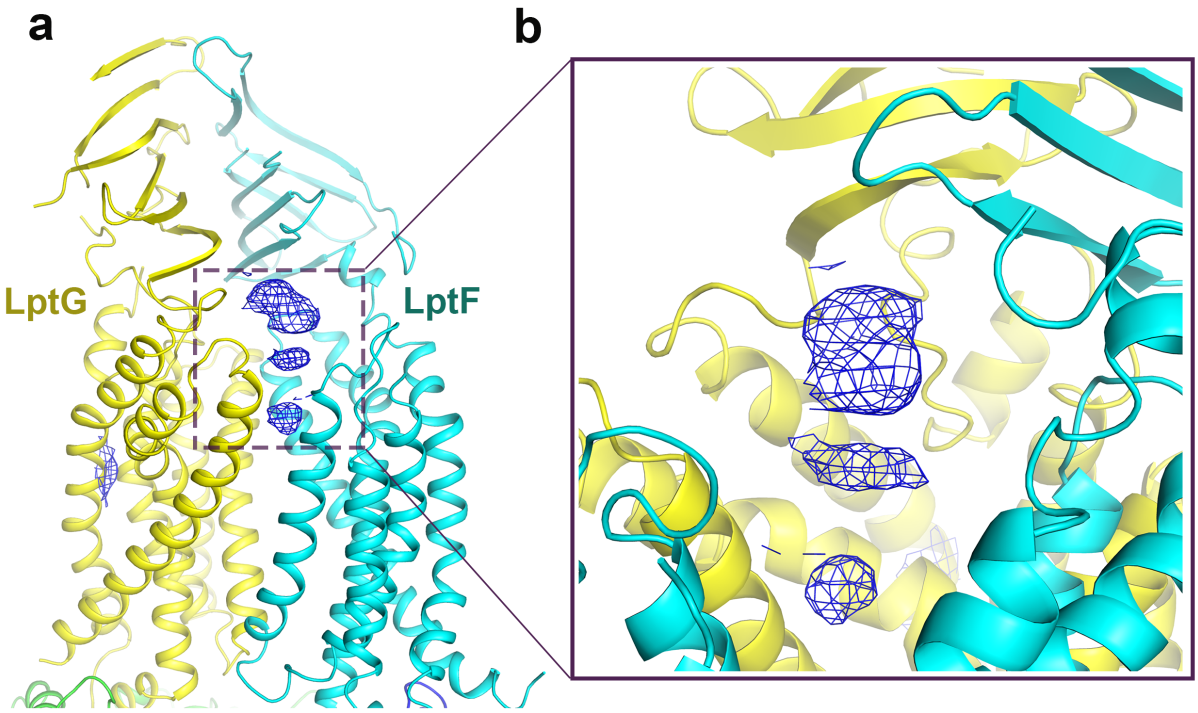

Supplementary Figure 9. Extra-electron densities in the cavity of $LptB_2FG$. The Fo-Fc electron density contoured at 2.5σ. a. The unassigned electron densities in the cavity of $LptB_2FG$. b. The close view of the electron densities.

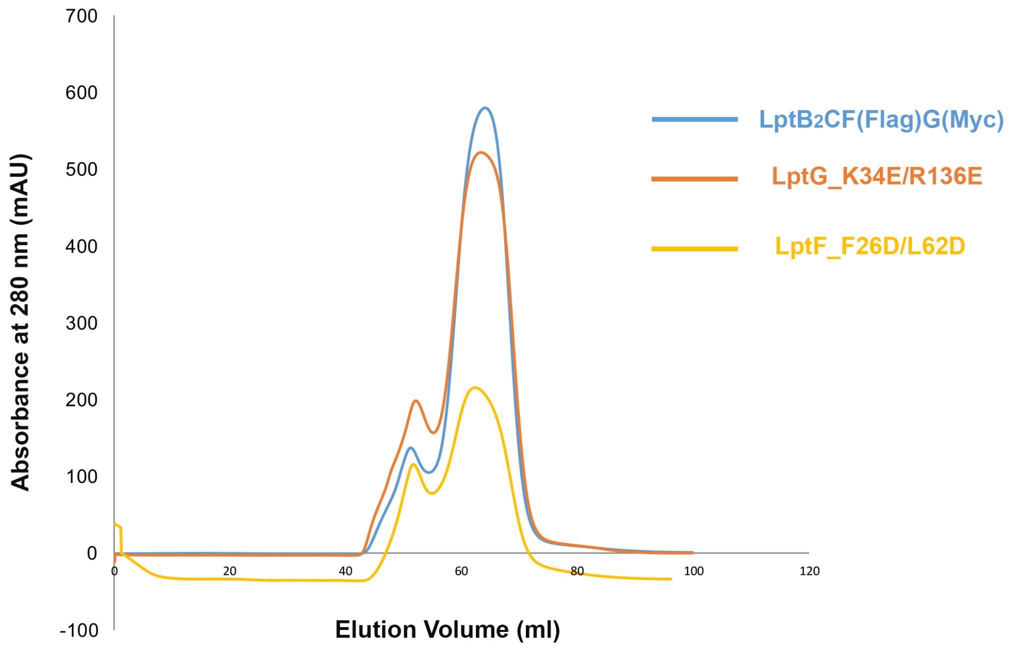

Supplementary Figure 10. Size-exclusion chromatography measurements of wild-type $LptB_2CFG$, $LptB_2CFG$ mutant LptF_F26D/L62D and $LptB_2CFG$ mutant LptG_K34E/R136E. *E. coli* wild-type $LptB_2CFG$, mutant LptF_F26D/L62D and mutant LptG_K34E/R136E were expressed, and the protein complexes were purified by a nickel column. The protein complexes were analysed by size-exclusion chromatography using a HiLoad 16/600 Superdex 200 pg column. The two mutant complexes were eluted at the same elution volume as that of the wild-type, suggesting that the two mutants are folded.

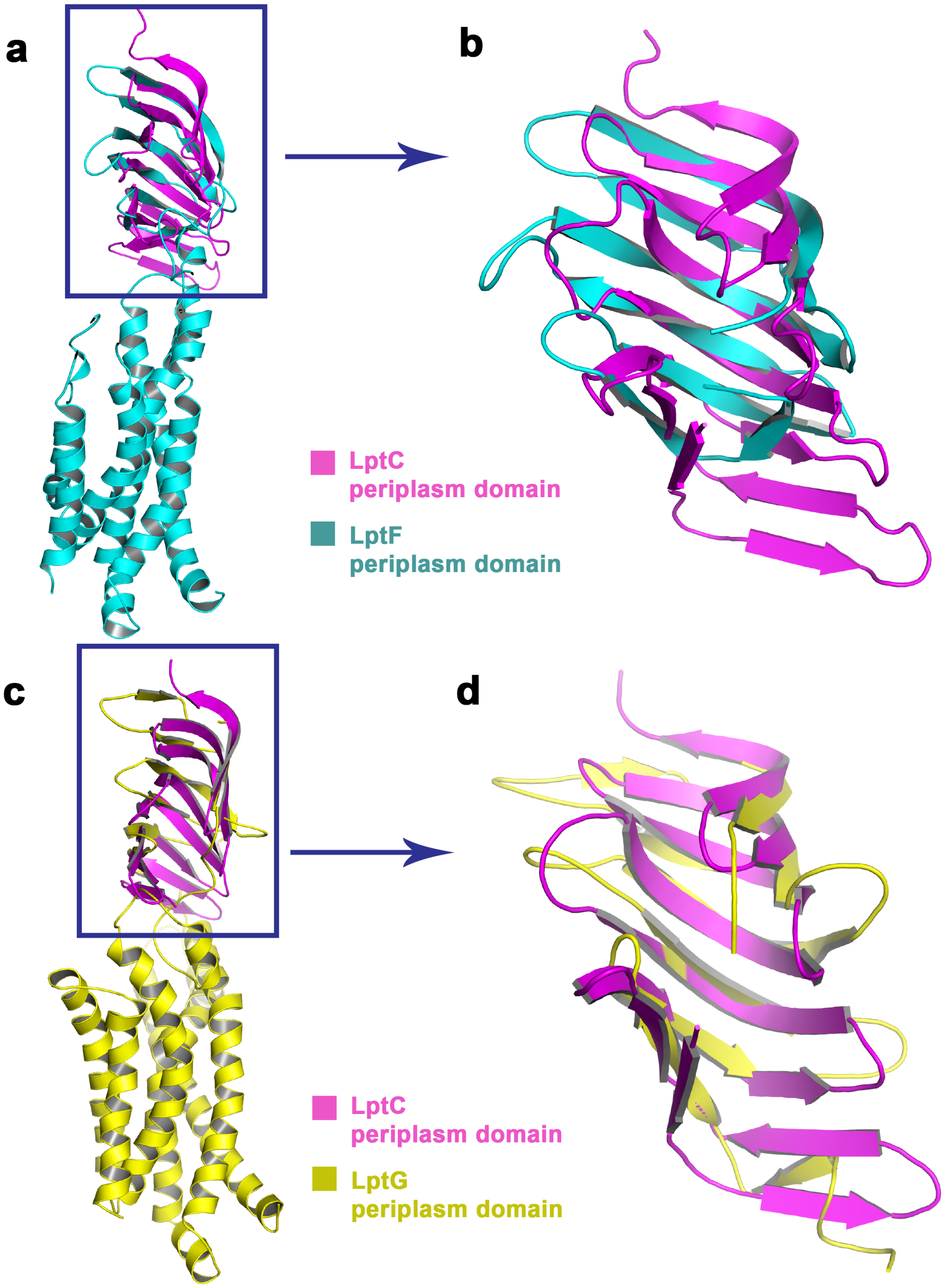

Supplementary Figure 11. Periplasmic domains of LptF and LptG resemble LptC. LptF, LptG and LptC molecules are in cyan, yellow and magenta, respectively. a. The periplasmic domain of LptC (PDB code 3MY2) is superimposed to the periplasmic domain of LptF. b. A close view of the superimposition of LptC and the periplasmic domain of LptF. c. The LptC is superimposed to the periplasmic domain of LptG. d. A close view of the superimposition of LptC and the periplasmic domain of LptG.

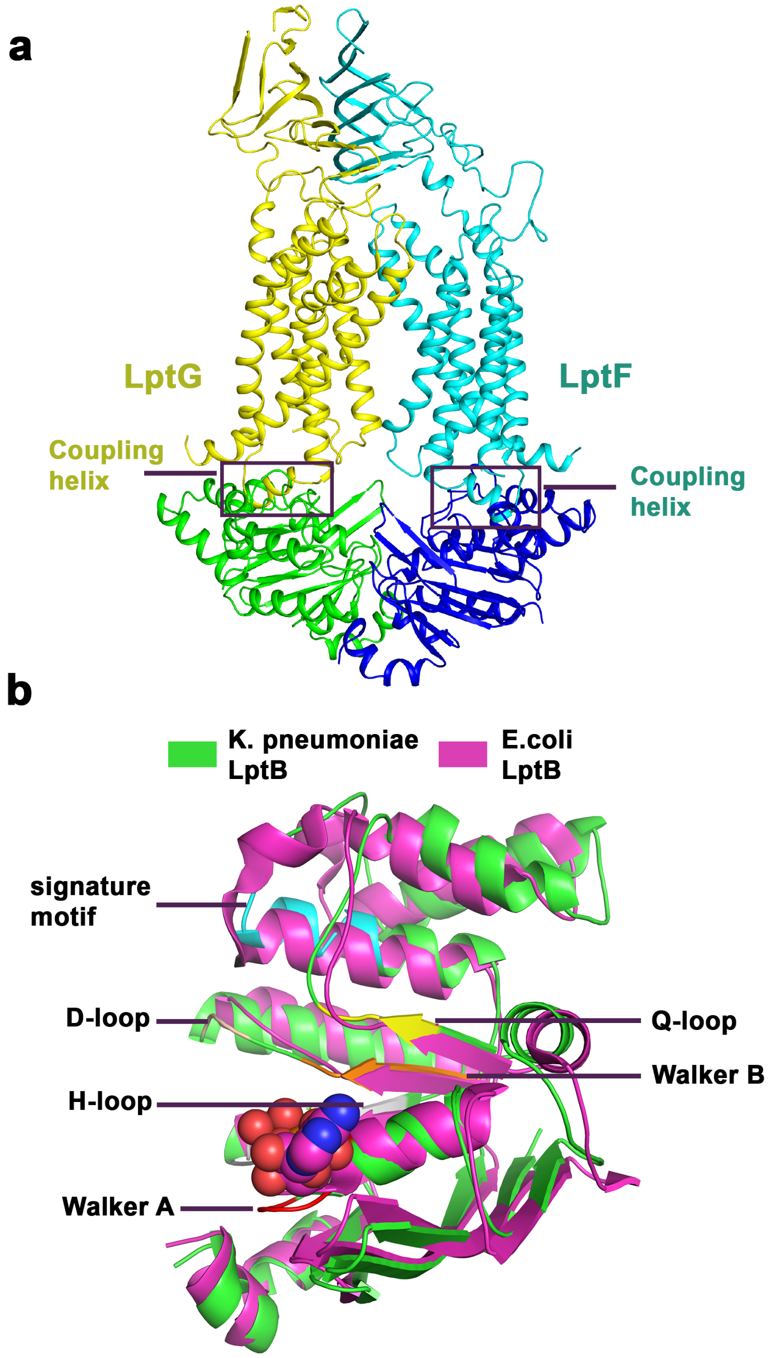

Supplementary Figure 12. LptB structures of the *E. coli* and *K. pneumoniae* and the coupling helices of LptF and LptG. LptB of *E. coli* was only crystallized in presence of nucleotide (ATP, ADP or AMP). The ATP-free LptB structure of *K. pneumoniae* was determined. a. The coupling helices of LptF and LptG. b. Superimposition of structures of *E. coli* LptB (magenta) (PDB code 4QC2) and *K. pneumoniae* LptB (green). The conformational changes were observed when ATP bound. The sequence identity of *E. coli* LptB and *K. pneumoniae* LptB is 95.85%.

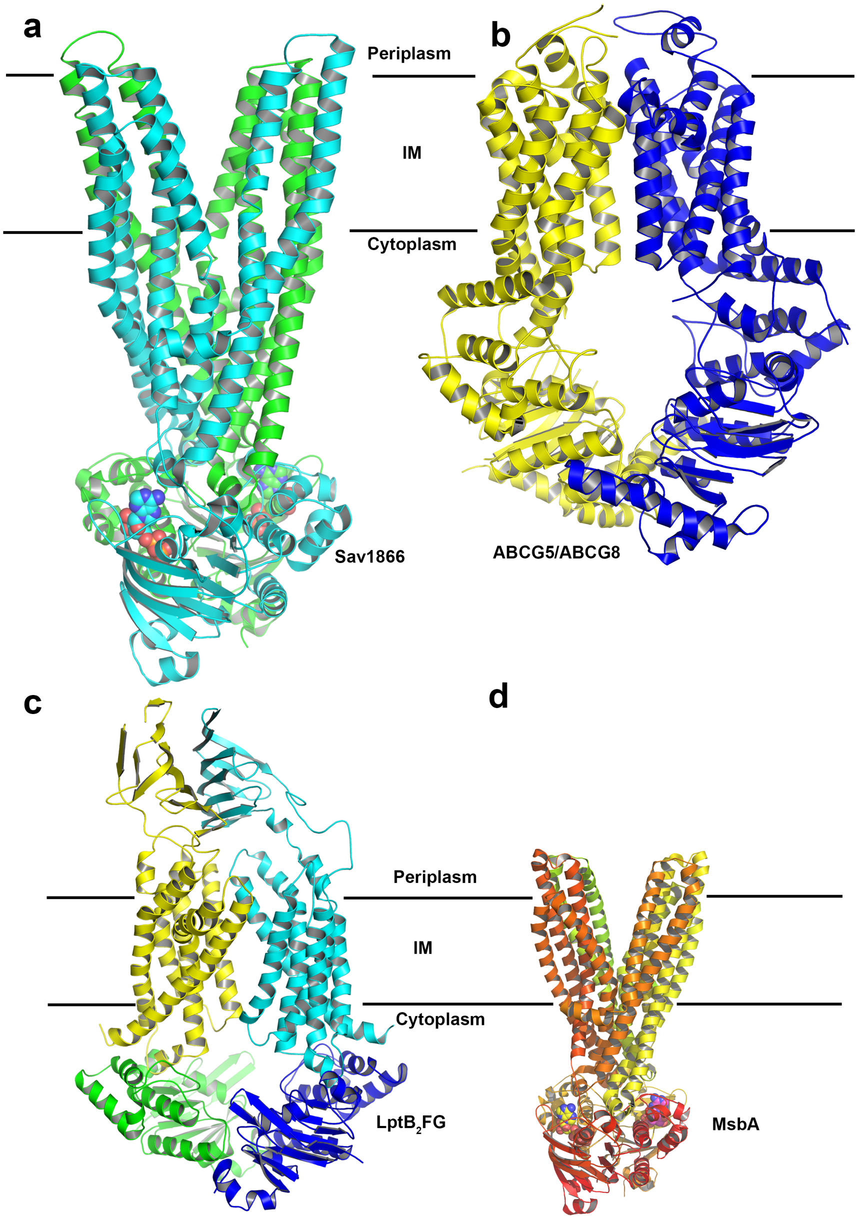

Supplementary Figure 13. Crystal structures of ABC transporters in different conformations. a. The multidrug transporter Sav1866 in complex with ADP is in an outward-facing conformation (PDB code 2HYD). The NBDs is in “close contact”. b. Crystal structure of human sterol transporter ABCG5/ABCG8 (PDB code 5DO7). The structure is in an inward-facing conformation. The NBDs are ATP-free and in “loose contact”. c. Crystal structure of $LptB_2FG$. The lateral gate TM5F-1G is in an open conformation, while the NBDs are in ATP-free and in “loose contact”. The ATP-binding and hydrolysis may fully open the lateral gate TM5F-1G and drive LPS transport. d. MsbA in complex with AMP-PNP is in the outward-facing conformation (PDB code 3B60).

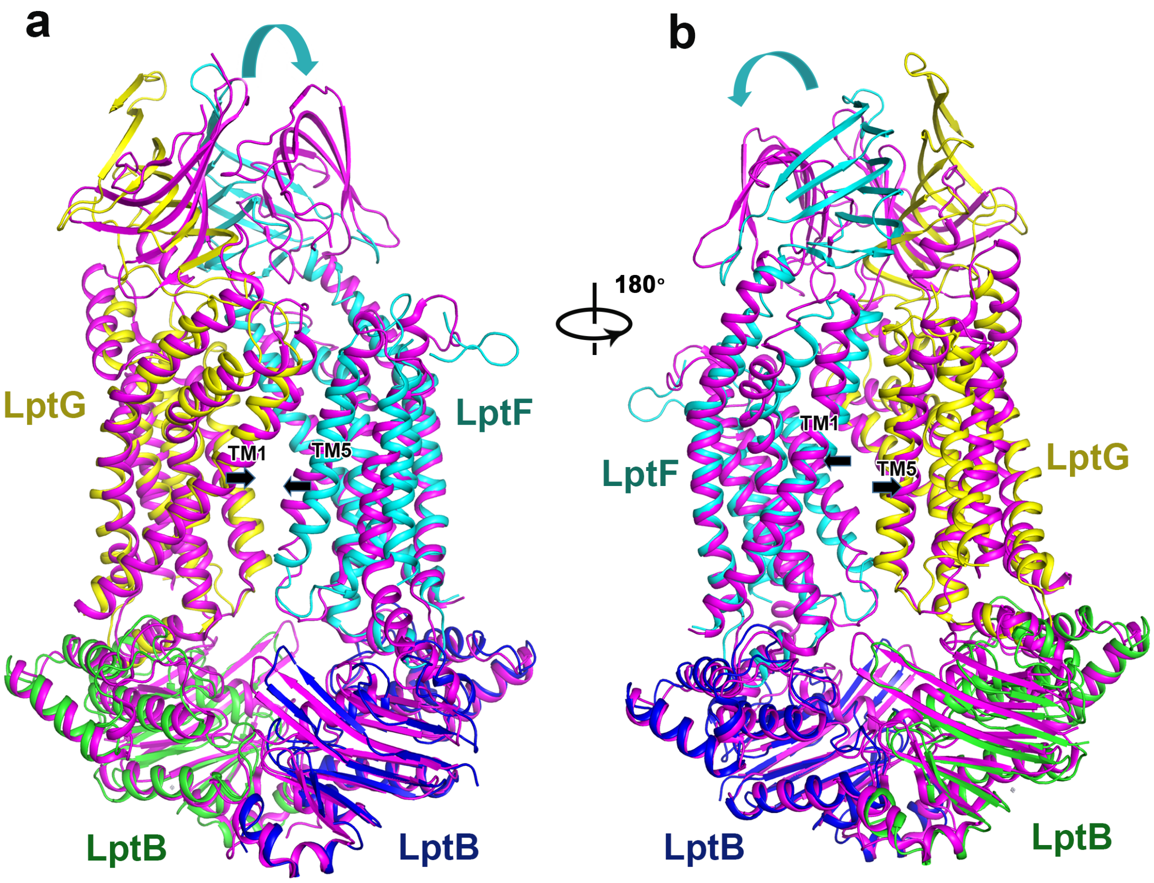

Supplementary Figure 14. The structure of LptB$_2$FG from *K. pneumoniae* is in a totally different conformation from that of LptB$_2$FG from *P. aeruginosa*. The colour scheme of LptB$_2$FG from *K. pneumoniae* is the same as that of the Fig 2 (LptF and LptG are in cyan and yellow, respectively). The LptB$_2$FG of *P. aeruginosa* is in magenta. The two transporter structures are superimposed. a. By comparing to the structure of LptB$_2$FG of *K. pneumonia*, the periplasmic domains of LptB$_2$FG from *P. aeruginosa* rotate around 90° toward to the lateral gate TM5F-TM1G, whereas the lateral gate TM5F-TM1G is from an open conformation (*K. pneumoniae*) to a closed conformation (*P. aeruginosa*). The arrows showed that the lateral gate TM5F-TM1G is from the open conformation to the closed conformation. b. By comparing to the structure of LptB$_2$FG of *K. pneumonia*, the conformations of the periplasmic domains of LptB$_2$FG of *P. aeruginosa* have changed, whereas the lateral gate TM1F-TM5G is from a closed conformation (*K. pneumoniae*) to an open conformation (*P. aeruginosa*). The arrows showed the conformational changes of the TM1F-TM5G.

```
                1         10        20        30        40        50        60
F_Shig          MIIIRYLVRETLKSQLAILFILLLIFFCQKLVRILGAAVDGDIPANLVLSLLGLGVPEMA
F_Ecoli         MIIIRYLVRETLKSQLAILFILLLIFFCQKLVRILGAAVDGDIPANLVLSLLGLGVPEMA
F_Kleb          MIIIRYLVRETLKSQLAILFILLLIFFCQKLVRILGAAVDGDIPTNLVLSLLGLGIPEMA
consensus>50    MIIIRYLVRETLKSQLAILFILLLIFFCQKLVRILGAAVDGDIPaNLVLSLLGLG!PEMA

                          70        80        90       100       110       120
F_Shig          QLILPLSLFLGLLMTLGKLYTESEITVMHACGLSKAVLVKAAMILAVFTAIVAAVNVMWA
F_Ecoli         QLILPLSLFLGLLMTLGKLYTESEITVMHACGLSKAVLVKAAMILAVFTAIVAAVNVMWA
F_Kleb          QLILPLSLFLGLLMTLGKLYTESEITVMHACGLSKAVLIKAAMILALFTGAVAAVNVMWA
consensus>50    QLILPLSLFLGLLMTLGKLYTESEITVMHACGLSKAVL!KAAMILAvFTaiVAAVNVMWA

                         130       140       150       160       170       180
F_Shig          GPWSSRHQDEVLAEAKANPGMAALAQGQFQQATNGSSVLFIESVDGSDFKDVFLAQIRPK
F_Ecoli         GPWSSRHQDEVLAEAKANPGMAALAQGQFQQATNGSSVLFIESVDGSDFKDVFLAQIRPK
F_Kleb          GPWSSRHQDEVLAEAKANPGMAALAQGQFQQASDGNAVMFIESVNGNRFHDVFLAQLRPK
consensus>50    GPWSSRHQDEVLAEAKANPGMAALAQGQFQQAt#GssV$FIESV#GsdFkDVFLAQiRPK

                         190       200       210       220       230       240
F_Shig          GNARPSVVVADSGHLTQLRDGSQVVTLNQGTRFEGTALLRDFRITDFQDYQAIIGHQAVA
F_Ecoli         GNARPSVVVADSGHLTQLRDGSQVVTLNQGTRFEGTALLRDFRITDFQDYQAIIGHQAVA
F_Kleb          GNARPSVVVADSGELSQQKDGSQVVTLNKGTRFEGTAMLRDFRITDFNNYQAIIGHQAVS
consensus>50    GNARPSVVVADSGhLtQlrDGSQVVTLNqGTRFEGTA$LRDFRITDF##YQAIIGHQAVa

                         250       260       270       280       290       300
F_Shig          LDPNDTDQMDMRTLWNTDTDRARAELNWRITLVVTVFMMALMVVPLSVVNPRQGRVLSML
F_Ecoli         LDPNDTDQMDMRTLWNTDTDRARAELNWRITLVFTVFMMALMVVPLSVVNPRQGRVLSML
F_Kleb          ADPDDTEQMDMRTLWKTHTDRARAELHWRFTLVATVFIMALMVVPLSVVNPRQGRVLSML
consensus>50    lDP#DT#QMDMRTLWnTdTDRARAELnWRiTLV.TVFmMALMVVPLSVVNPRQGRVLSML

                         310       320       330       340       350       360
F_Shig          PAMLLYLLFFLIQTSLKSNGGKGKLDPTLWMWTVNLIYLALAIVLNLWDTVPVRRLRASF
F_Ecoli         PAMLLYLLFFLIQTSLKSNGGKGKLDPTLWMWTVNLIYLALAIVLNLWDTVPVRRLRASF
F_Kleb          PAMLLYLVFFLLQTSIKSNGGKGKMDPAIWMWAINLLYFALAVLLNLWDTVPMRRFRARF
consensus>50    PAMLLYLlFFLiQTSlKSNGGKGK$DPtlWMWt!NLiYlALA!vLNLWDTVPvRRlRAsF

F_Shig          SRKGAV
F_Ecoli         SRKGAV
F_Kleb          N.KGAA
consensus>50    srKGAv
```

Supplementary Figure 15. The sequence alignment of LptF from *S. flexneri* (Shig*)*, *E. coli* (Ecoli) and *K. pneumonia* (Kleb). The LptF amino acid sequences from these three species are highly conserved. The bottom is the consensus sequence (Consensus > 50). Uppercase is identity; lowercase is consensus level >0.5; ! is anyone of IV; $ is anyone of LM; % is anyone of FY; # is anyone of NDQEBZ.

```
                 1        10        20        30        40        50        60
G_Shig           MQPFGVLDRYIGKTIFTTIMMTLFMLVSLSGIIKFVDQLKKAGQGSYDALGAGMYTLLSV
G_Ecoli          MQPFGVLDRYIGKTIFTTIMMTLFMLVSLSGIIKFVDQLKKAGQGSYDALGAGMYTLLSV
G_Kleb           MQAFGVLDRYIGKTIFNTIMMTLFMLVSLSGIIKFVDQLKKSGQGSYDALGAGLYTILSV
consensus>50     MQpFGVLDRYIGKTIFtTIMMTLFMLVSLSGIIKFVDQLKKaGQGSYDALGAG$YTlLSV

                          70        80        90       100       110       120
G_Shig           PKDVQIFFPMAALLGALLGLGMLAQRSELVVMQASGFTRMQVALSVMKTAIPLVLLTMAI
G_Ecoli          PKDVQIFFPMAALLGALLGLGMLAQRSELVVMQASGFTRMQVALSVMKTAIPLVLLTMAI
G_Kleb           PKDIQIFFPMAALLGALLGLGMLAQRSELVVMQASGFTRLQVALAVMKTAIPLVLLTMAI
consensus>50     PKD!QIFFPMAALLGALLGLGMLAQRSELVVMQASGFTR$QVALsVMKTAIPLVLLTMAI

                         130       140       150       160       170       180
G_Shig           GEWVAPQGEQMARNYRAQAMYGGSLLSTQQGLWAKDGNNFVYIERVKGDEVLGGISIYAF
G_Ecoli          GEWVAPQGEQMARNYRAQAMYGGSLLSTQQGLWAKDGNNFVYIERVKGDEELGGISIYAF
G_Kleb           GEWVAPQGEQMARNYRAQQMYGGSLLSTQQGLWAKDGHNFVYIERVKGNDELGGVSIYAF
consensus>50     GEWVAPQGEQMARNYRAQaMYGGSLLSTQQGLWAKDGnNFVYIERVKG##eLGG!SIYAF

                         190       200       210       220       230       240
G_Shig           NENRRLQSVRYAATAKFDPEHKVWRLSQVDESDLTNPKQITGSQTVSGTWKTDLTPDKLG
G_Ecoli          NENRRLQSVRYAATAKFDPEHKVWRLSQVDESDLTNPKQITGSQTVSGTWKTNLTPDKLG
G_Kleb           NPERRLQSVRYAASAKFDSENKVWRLSQVDESDLTDPKQVTGSQMVSGTWKTNLTPDKLG
consensus>50     Ne#RRLQSVRYAAtAKFDpEhKVWRLSQVDESDLT#PKQ!TGSQtVSGTWKT#LTPDKLG

                         250       260       270       280       290       300
G_Shig           VVALDPDALSISGLHNYVKYLKSSGQDAGRYQLNMWSKIFQPLSVAVMMLMALSFIFGPL
G_Ecoli          VVALDPDALSISGLHNYVKYLKSSGQDAGRYQLNMWSKIFQPLSVAVMMLMALSFIFGPL
G_Kleb           VVALDPDALSISGLHNYVKYLKSSGQDPGRYQLNMWSKIFQPLSVAVMMLMALSFIFGPL
consensus>50     VVALDPDALSISGLHNYVKYLKSSGQDaGRYQLNMWSKIFQPLSVAVMMLMALSFIFGPL

                         310       320       330       340       350       360
G_Shig           RSVPMGVRVVTGISFGFVFYVLDQIFGPLTLVYGIPPIIGALLPSASFFLISLWLLMRKS
G_Ecoli          RSVPMGVRVVTGISFGFVFYVLDQIFGPLTLVYGIPPIIGALLPSASFFLISLWLLMRKS
G_Kleb           RSVPMGVRVVTGISFGFIFYVLDQIFGPLTLVYGIPPIIGALLPSASFFLISLWLMMRKA
consensus>50     RSVPMGVRVVTGISFGF!FYVLDQIFGPLTLVYGIPPIIGALLPSASFFLISLWL$MRKs
```

Supplementary Figure 16. The sequence alignment of LptG from *S. flexneri* (Shig*)*, *E. coli* (Ecoli), and *K. pneumonia* (Kleb). The LptG amino acid sequences from these three species are highly conserved. The bottom is the consensus sequence (Consensus > 50). Uppercase is identity; lowercase is consensus level >0.5; ! is anyone of IV; $ is anyone of LM; % is anyone of FY; # is anyone of NDQEBZ.

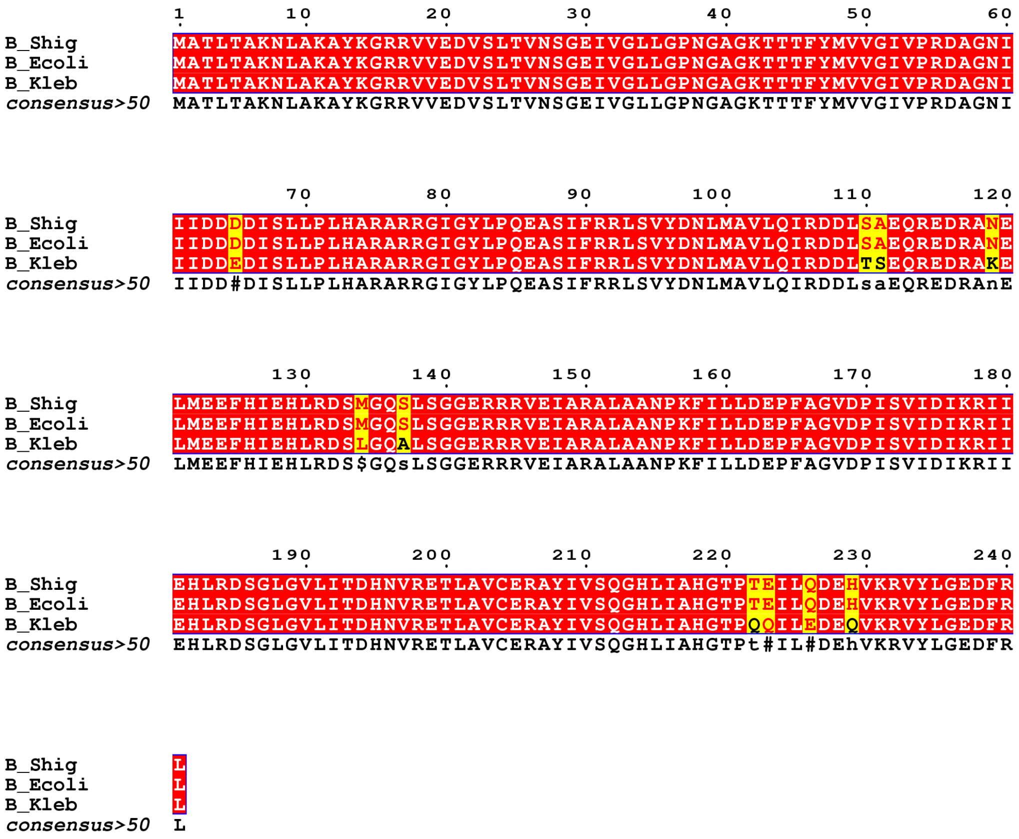

Supplementary Figure 17. The sequence alignment of LptB from *S. flexneri* (Shig*)*, *E. coli* (Ecoli), and *K. pneumonia* (Kleb). The LptB amino acid sequences from these three species are highly conserved. The bottom is the consensus sequence (Consensus > 50). Uppercase is identity; lowercase is consensus level >0.5; ! is anyone of IV; $ is anyone of LM; % is anyone of FY; # is anyone of NDQEBZ.

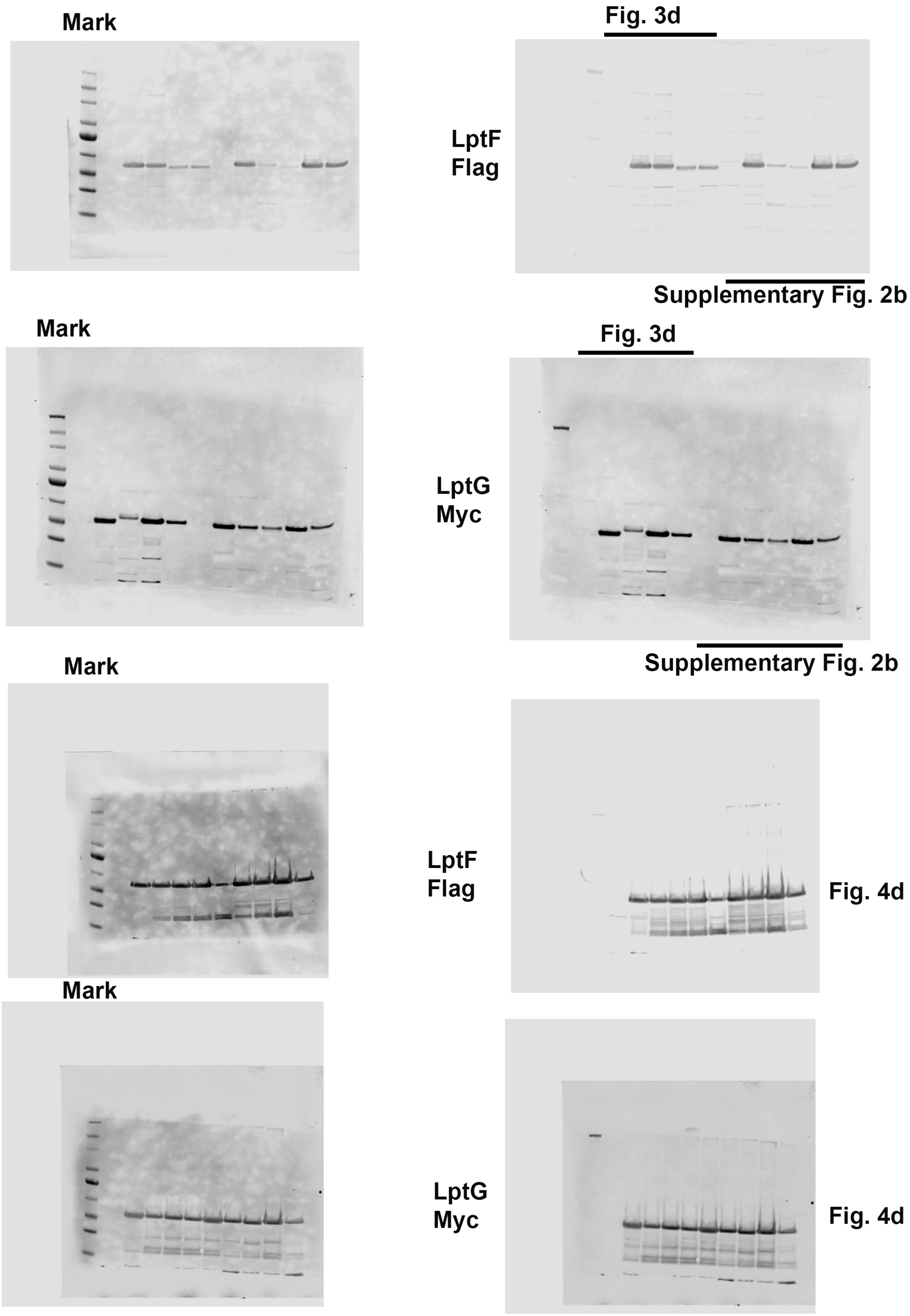

Supplementary Figure 18. The original western blots of Figure 3d, supplementary Figure 2b and Figure 4d.

Supplementary Table 1. Primers used for generating constructions.

Shigella_lptB_EcoRI_F	ATATGAATTCATGGCAACATTAACTGCAAAGAACCTTGC
Shigella_lptB_KpnI_8.HIS_R	ATATGGTACCTCAGTGATGGTGATGGTGATGGTGATGGAGTCTGAAG TCTTCCCCAAGGTATACAC
Shigella_lptFG_KpnI_F	ATAT GGTACC TTTTTACGGGCGTATTTAAAGTGATAATC
Shigella_lptFG_XbaI_R	ATAT TCTAGA TTACGATTTTCTCATTAACAGCCACAG
Klebsiella_lptB_EcoRI_F	ATAT GAATTC ATGGCAACCTTAACTGCGAAGAATCTCG
Klebsiella_lptB_KpnI_8.HIS_R	ATAT GGTACC TCA GTGATGGTGATGGTGATGGTGATG GAGTCTGAAGTCTTCCCCAAG
Klebsiella_lptFG_KpnI_F	ATATGGTACCTTTTTACGGGCGTATTTATAGTGATAATCATAAGATATC TGGTTCG
Klebsiella_lptFG_XbaI_R	ATAT TCTAGA TTA GGCCTTGCGCATCATCAGCC
K12_F230_Flag_F	GATTACAAAGATGACGACGATAAA CAGGCGATCATTGGTCACCAGGC
K12_F230_Flag_R	TTTATCGTCGTCATCTTTGTAATCATAATCCTGGAAGTCCGTAATGCG GAAATCACG
K12_G228_Myc_F	GAACAAAAACTCATCTCAGAAGAGGATCTG ACCTGGAAAACCAACCT CACGCC
K12_G228_Myc_R	CCTCTTCTGAGATGAGTTTTTGTTC GCCGCTCACCGTCTGCGAAC
K12_F138_Flag_F	GATTACAAAGATGACGACGATAAA CCTGGCATGGCGGCGCTG
K12_F138_Flag_R	TTTATCGTCGTCATCTTTGTAATC GTTCGCTTTCGCTTCTGCTAACAC TTCATCCTG
K12_G144_Myc_F	GAACAAAAACTCATCTCAGAAGAGGATCTG TTGCTCTCTACCCAGCA AGGCTTATGG
K12_G144_Myc_R	CCTCTTCTGAGATGAGTTTTTGTTC CGAGCCGCCGTACATCGCC

Supplementary Table 2. Primers used for mutagenesis.

G_K34E_F	GGCATTATC GAA TTTGTCGAT CAGCTGAAAAAAGCCGGGCAGG
G_K34E_R	ATCGACAAA TTC GATAATGCC CGACAGCGACACCAGCATGAAC
G_R136E_F	GTAACTACGAAGCGCAG GCGATGTACGGCGGCTCG
G_R136E_R	CTGCGCTTCGTAGTTAC GCGCCATCTGCTCGCC
G_K40E_K41E_F	ATCAGCTGGAAGAAGCCG GGCAGGGGAGTTACGACGCG
G_K40E_K41E_R	CGGCTTCTTCCAGCTGAT CGACAAACTTGATAATGCCCGACAGCG
F_F26D_F	CTTTTGATCGATTTCTGTCAAAAGT TAGTGAGGATCCTCGGCGCAGC
F_F26D_R	ACTTTTGACAGAAATCGATCAAAAG CAAGATGAAGAGTATCGCCAGCT GGC
F_L62D_F	AAATGGCGCAGGATATCCT GCCATTAAGCCTGTTCCTCGGG
F_L62D_R	AGGATATCCTGCGCCATTT CCGGCACGCCCAACCC
F_D229P_F	TTCCAGCCGTATCAGG CGATCATTGGTCACCAGGCGGTG
F_D229P_R	CCTGATACGGCTGGAA GTCCGTAATGCGGAAATCACGTAACAATG
F_Q231P_F	GGATTATCCGGCGATCAT TGGTCACCAGGCGGTGGCGCTC
F_Q231P_R	ATGATCGCCGGATAATCC TGGAAGTCCGTAATGCGGAAATCACG
F_R223P_F	TGATTTCCCGATTACGGA CTTCCAGGATTATCAGGCGATCATTGGTC
F_R223P_R	TCCGTAATCGGGAAATCA CGTAACAATGCAGTGCCTTCGAAGC
F_T225P_F	TCCGCATTCCGGACT TCCAGGATTATCAGGCGATCATTGGTC
F_T225P_R	AGTCCGGAATGCGGA AATCACGTAACAATGCAGTGCCTTCG
G_S223P_F	TTACCGGTCCGCAGAC GGTGAGCGGCACCTGGAAAACC
G_S223P_R	GTCTGCGGACCGGTAATCTGTTTCGGATTGGTCAGATCAGATTCATCA AC
G_T225P_F	TTCGCAGCGGGTGA GCGGCACCTGGAAAACCAACCTC
G_T225P_R	TCACCCGCTGCGAA CCGGTAATCTGTTTCGGATTGGTCAGATC
G_G228P_F	GCCCGACCTGGAAAA CCAACCTCACGCCGGACAAAC
G_G228P_R	TTTTCCAGGTCGGGC ATCTGACCAATCCGAAACAGATTACCGGTTC
G_W230P_F	GCACCCGGAAAACCAA CCTCACGCCGGACAAACTGG
G_W230P_R	TTGGTTTTCCGGGTGC CGCTCACCGTCTGCGAACCGG
F_D229P_Q231P_F	AGCCGTATCCGGCG ATCATTGGTCACCAGGCGGTGG
F_D229P_Q231P_R	CGCCGGATACGGCT GGAAGTCCGTAATGCGGAAATCACGTAAC
F_R223P_T225P_F	TTTCCCGATTCCGGACTTC CAGGATTATCAGGCGATCATTGGTCACCAG
F_R223P_T225P_R	GAAGTCCGGAATCGGGAAA TCACGTAACAATGCAGTGCCTTCGAAG
G_S223P_T225P_F	GTCCGCAGCCGGTGAG CGGCACCTGGAAAACCAACCTCAC
G_S223P_T225P_R	CTCACCGGCTGCGGAC CGGTAATCTGTTTCGGATTGGTCAGATCAGATTC
G_G228P_W230P_F	AGCCCGACCCCGAAAA CCAACCTCACGCCGGACAAAC
G_G228P_W230P_R	TTTTCGGGGTCGGGCT CACCGTCTGCGAACCGGTAATCTG
